# Anthropogenic iron alters the spring phytoplankton bloom in the North Pacific transition zone

**DOI:** 10.1073/pnas.2418201122

**Published:** 2025-06-02

**Authors:** Nicholas J. Hawco, Tim M. Conway, Sacha N. Coesel, Benedetto Barone, Emily A. Seelen, Shun-Chung Yang, Randelle M. Bundy, Paulina Pinedo-Gonzalez, Xiaopeng Bian, Matthias Sieber, Nathan T. Lanning, Jessica N. Fitzsimmons, Rhea K. Foreman, Daniela König, Mora J. Groussman, James G. Allen, Lauren W. Juranek, Angelicque E. White, David M. Karl, E. Virginia Armbrust, Seth G. John

**Affiliations:** ^a^Department of Oceanography, University of Hawai’i at Mānoa, Honolulu, HI 96822; ^b^College of Marine Science, University of South Florida, St. Petersburg, FL 33701; ^c^School of Oceanography, University of Washington, Seattle, WA 98195; ^d^Department of Earth Sciences, University of Southern California, Los Angeles, CA 90089; ^e^College of Fisheries and Ocean Sciences, University of Alaska Fairbanks, Fairbanks, AK 99775; ^f^Department of Oceanography, Texas A&M University, College Station, TX 77843; ^g^College of Earth, Ocean, and Atmospheric Sciences, Oregon State University, Corvallis, OR 97331

**Keywords:** nutrient limitation, atmospheric deposition, biogeochemical cycles, GEOTRACES, gradients

## Abstract

Industrial activities have increased the supply of iron to the ocean, but the magnitude of anthropogenic input and its ecological consequences are not well-constrained by observations. Across four expeditions to the North Pacific transition zone, we document a repeated supply of isotopically light iron from an atmospheric source in spring, reflecting an estimated 39 ± 9 % anthropogenic contribution to the surface ocean iron budget. Expression of iron-stress genes in metatranscriptomes, and evidence for colimitation of ecosystem productivity by iron and nitrogen, indicates that enhanced iron supply should spur spring phytoplankton blooms, accelerating the seasonal drawdown of nitrate delivered by winter mixing. This effect is consistent with regional trends in satellite ocean color, which show a shorter, more intense spring bloom period, followed by an earlier arrival of oligotrophic conditions in summer. Continued iron emissions may contribute to poleward shifts in transitional marine ecosystems, compounding the anticipated impacts from ocean warming and stratification.

## Significance

Iron is an essential nutrient for phytoplankton photosynthesis, but its low solubility in seawater makes large regions prone to iron limitation, a state where exogenous iron input can increase photosynthesis, primary production, and carbon export. Industrial emissions now add to the natural iron supply, but ecosystem impacts from anthropogenic iron input have not yet been demonstrated. Here, we focus on the North Pacific transition zone, a seasonally iron-deficient biome downwind from major industrial centers in East Asia. Over the past 25 y, increasing anthropogenic iron input appears to have stimulated springtime phytoplankton growth, ultimately leading to faster depletion of other nutrients, especially nitrate. Thus, large-scale iron pollution may be increasing the prevalence of nitrogen limitation, expanding the oligotrophic ocean.

Relative to the preindustrial period, aerosol emissions from metal smelting, steel production, coal combustion, and other activities are thought to have dramatically increased levels of soluble iron (Fe) in atmospheric aerosols (1, 2). Fe originating from these anthropogenic sources (*Fe_anthro_*)—traceable by the light Fe isotope signature associated with high temperature volatilization—has been detected in urban particulate matter and in aerosols over the remote Pacific and Atlantic Oceans (3–5). Experiments also indicate that *Fe_anthro_* dissolves more readily than desert dust— the primary natural source of dissolved Fe (dFe) to the surface ocean (3, 5, 6). Because dFe is scarce across remote oceanic biomes, several modeling studies have indicated that *Fe_anthro_* supply may now occur at sufficient scale to influence oceanic primary production, nitrogen fixation, and other processes that are often limited by natural Fe deficiency (7). While these model projections await observational confirmation, past sampling has documented substantial accumulations of anthropogenic nitrogen and lead in the central and western Pacific, which derive, in part, from similar sources (8, 9).

**Fig. 1.**
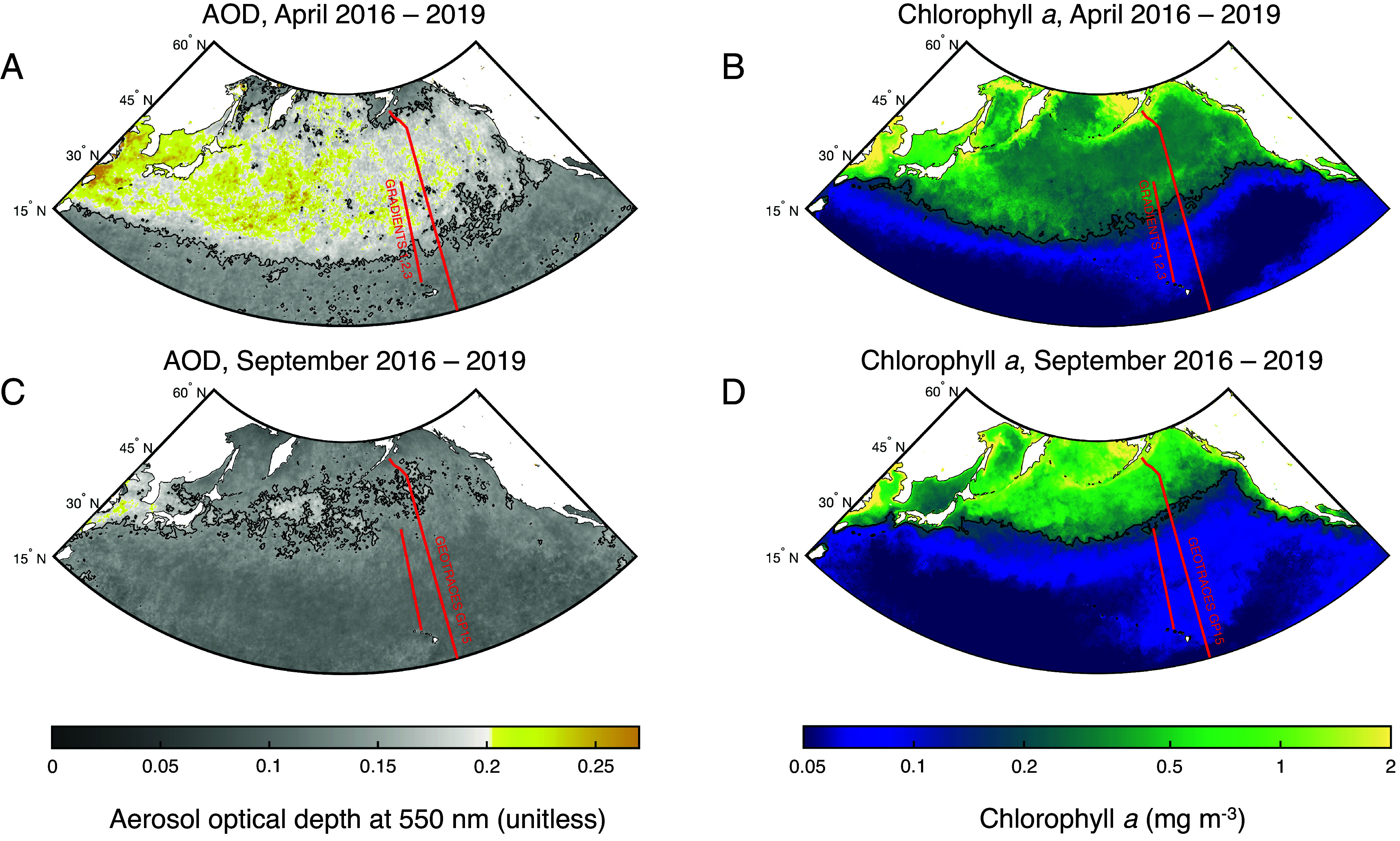
Seasonal shifts in satellite-detected aerosol optical depth (AOD, unitless) and Chlorophyll *a* (mg m^−3^, log scale) across the North Pacific. Distributions of the 2016 to 2019 mean AOD and Chlorophyll *a* for April (*A* and *B*) and September (*C* and *D*) are shown. In all plots, red lines indicate transects for the Gradients cruises in 2016, 2017, and 2019 along 158 °W and the GEOTRACES GP15 transect, following 152 °W. Contours at AOD = 0.1 (*A* and *C*) approximately distinguish continental aerosols from background marine aerosols. Elevated AOD in April (*A*) reflects dust and anthropogenic aerosols originating from Asia, which declines by September (*C*). Contours in (*B* and *D*) follow the 0.2 mg m^−3^ transition zone chlorophyll front (TZCF), which extends farther south in April.

Since 2000, the bulk of *Fe_anthro_* emissions globally have originated from East Asia (10) and are subsequently carried out to sea by the westerly winds (Fig. 1). Initial mass balance calculations based on the Fe isotope composition (δ^56^Fe) of dFe in surface waters have suggested that *Fe_anthro_* could contribute up to half of the Fe supply to the North Pacific transition zone (NPTZ) (11), a highly productive biogeochemical province between the subtropical and subpolar gyres (approximately 30 to 45 °N) that plays an important role in the foraging ecology of pelagic animals (12, 13). However, the underconstrained fractionation of δ^56^Fe in seawater by biological processes makes these attributions uncertain (14). Here, we present an expanded dataset of δ^56^Fe in surface waters that confirms the importance of springtime *Fe_anthro_* supply to the NPTZ and its subsequent alteration throughout the seasonal cycle. Based on regional trends in satellite-detected ocean color and molecular evidence indicating that phytoplankton in the NPTZ are Fe-deficient during spring, we propose that the *Fe_anthro_* flux to the North Pacific is leading to an earlier arrival of nutrient-deplete, summertime conditions, contributing to a northward shift in biogeochemical provinces.

## Results and Discussion

At present, timeseries observations of the ocean’s Fe cycle are rare and have largely focused on accessible locations within reach of continental and island ports (15–17). Time-resolved measurements of δ^56^Fe are even more limited. To construct an apparent seasonal cycle of dFe and δ^56^Fe, we combined data from four expeditions across the remote NPTZ between 2016 and 2019 (Fig. 1). Three springtime transects were undertaken along 158 °W by the Gradients program in 2016, 2017, and 2019 (18). The GEOTRACES GP15 expedition was completed during Sept.–Oct. 2018 along 152 °W. To minimize the impact of mesoscale patchiness in ocean mixing and atmospheric deposition on the seasonal cycle, dFe and δ^56^Fe from these transects were grouped within coarse (~4°) latitudinal bins and averaged (*SI Appendix*, Fig. S1).

**Fig. 2.**
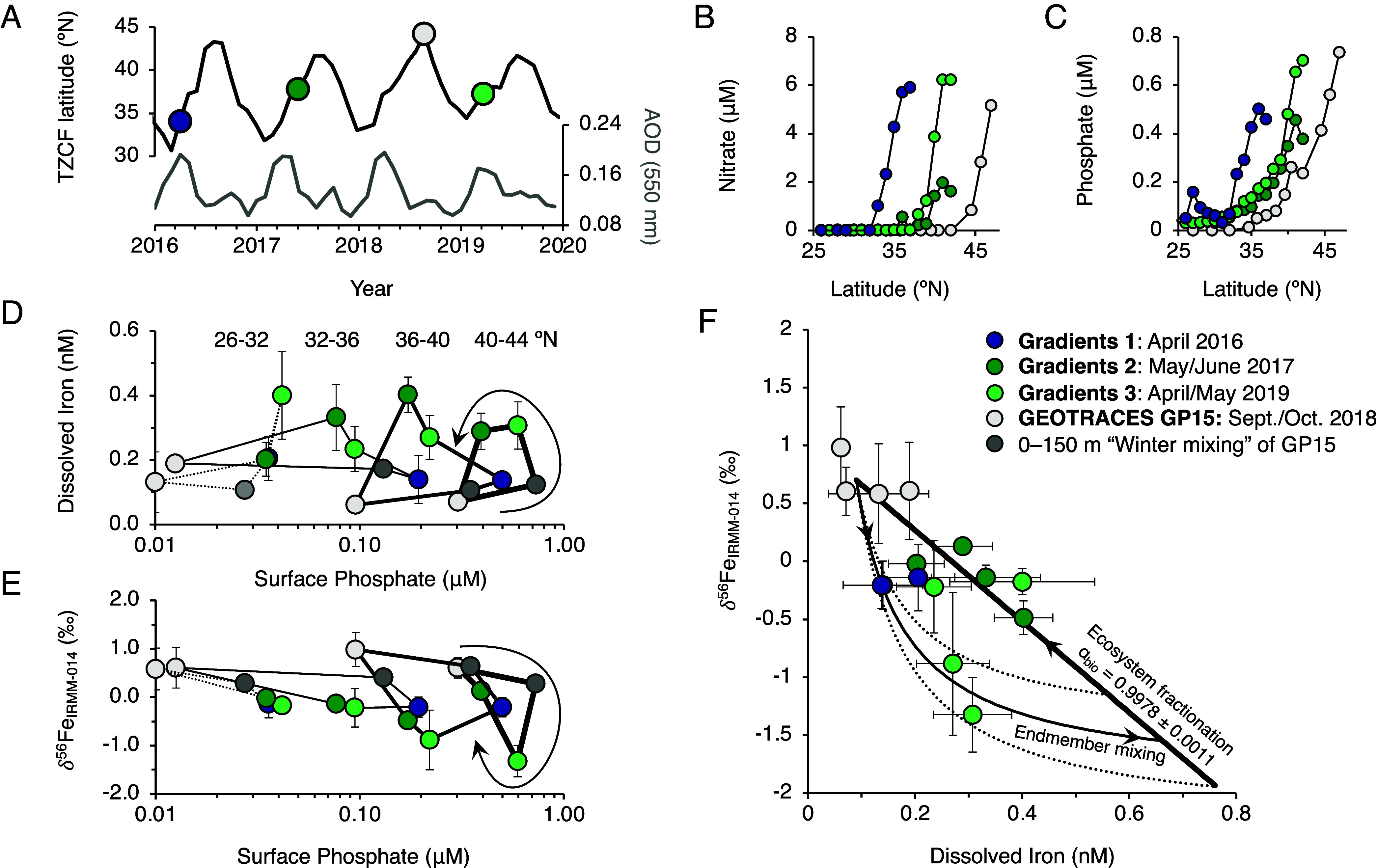
Seasonal evolution of dissolved Fe and Fe isotope ratios (δ^56^Fe) in the NPTZ, based on 4 expeditions between 2016 to 2019. (*A*) Latitude of the TZCF, with expeditions shown in circles (color code is shared across all panels). Gray line shows monthly aerosol optical depth (AOD) over the NPTZ region. Latitudinal distributions of macronutrients (*B*) nitrate and (*C*) phosphate across all four expeditions showing increasing nutrient depletion as the seasonal cycle progresses. Patterns in latitudinally binned (*D*) dFe and (*E*) δ^56^Fe, connected by lines of equal thickness, as a function of surface phosphate concentration, show supply of isotopically light Fe in spring (due to significant production of dissolved organic nitrogen, surface phosphate appears to be a more robust tracer of nutrient export, see *SI Appendix*, Fig. S1). Dark gray circles show the alteration of dFe and δ^56^Fe upon mixing 0 to 150 m of the autumn water column, based on the GP15 dataset (see *SI Appendix*, Fig. S2 for sensitivity analysis). (*F*) Concentration-isotope plot of latitudinally binned data. Black line shows the best fit (combined R^2^ = 0.86, see *Materials and Methods*) of open-system biological fractionation (α_bio_ = 0.998 ± 0.001) and endmember isotope mixing between background seawater (dFe = 0.09 nM; δ^56^Fe _IRMM-014_ = +0.70 ‰) and an atmospheric source (δ^56^Fe_IRMM-014_ = –1.90 ± 0.40 ‰).

During spring, shifting wind patterns lead to pulses of continental aerosols across the North Pacific, a time when the NPTZ is undergoing an annual biogeochemical transformation (Fig. 1). Nitrate (NO_3_^–^) and phosphate (PO_4_^3–^) supplied by convective mixing and southward wind-driven (Ekman) transport fuel productive waters and elevated chlorophyll across the NPTZ, which persist until phytoplankton exhaust NO_3_^–^ stocks in the surface mixed layer (12, 19). Due, in part, to the meridional gradient in NO_3_^–^ supply, the transition to oligotrophic conditions arrives sooner at more southern latitudes, causing the boundary between high and low productivity regimes—the transition zone chlorophyll front (TZCF), defined here at 0.2 mg chlorophyll *a* m^−3^—to migrate from 33 °N in February to 42 °N in August (Fig. 2*A*), a distance exceeding 1,000 km (12). Interannual variability in the timing of this seasonal shift was apparent in our shipboard observations; we crossed the TZCF at 34 °N in April 2016 and 37 °N in April 2019 (18). However, many of the biogeochemical differences between the springtime transects could be accounted for by the nutrient content of the surface mixed layer (Fig. 2 *B* and *C*) and thus reflect differences in the extent of the same cycle of nutrient depletion by spring phytoplankton blooms.

When plotted against the nutrient content of the surface mixed-layer, a clear seasonal cycle in dFe and δ^56^Fe emerged (Fig. 2 *D* and *E*). In autumn (Oct. 2018), NO_3_^–^ and PO_4_^3–^ were maximally depleted and dFe in the mixed layer was very low and isotopically heavy (32 to 44 °N mean: dFe = 0.14 ± 0.11 nM, δ^56^Fe = +0.63 ± 0.15 ‰, 1 SD). Only minor changes in dFe were observed through the upper 300 m of the water column (*SI Appendix*, Fig. S2). Across the NPTZ, winter mixed layer depths are typically 100 to 200 m (*SI Appendix*, Figs. S2 and S3), which is deep enough to entrain considerable NO_3_^–^ and PO_4_^3–^ from the subsurface. Indeed, high NO_3_^–^ and PO_4_^3–^ concentrations (>1 and >0.2 μM, respectively) were observed throughout the NPTZ in April 2016, when the TZCF was positioned farthest south. Meanwhile, dFe remained relatively low in April 2016 (0.14 ± 0.06 nM). Subsequent spring transects found lower NO_3_^–^ and PO_4_^3–^, increased dFe (0.35 ± 0.09 nM in 2017 and 0.26 ± 0.07 nM in 2019) and lighter isotopic compositions (δ^56^Fe = –0.28 ± 0.26 ‰ and –0.71 ± 0.63 ‰, respectively; Fig. 2 *D* and *E*) than observed in autumn (ANOVA with post hoc Tukey test: *P* < 0.05; see *SI Appendix*, Fig. S3).

Winter mixing alone cannot account for dFe and δ^56^Fe observed in the surface mixed layer during spring. If the impact of winter mixing is simulated by homogenizing the upper 150 m of the water column observed in autumn 2018 (calculated as the 0 to 150 m depth integral of dFe and the concentration-weighted δ^56^Fe, divided by the integration depth), the dFe concentration in the mixed layer would not exceed 0.2 nM, nor would δ^56^Fe fall below 0 ‰ (dark gray circles in Fig. 2 *D* and *E*). In the ocean’s subsurface, dFe is continually subject to bacterial uptake and scavenging onto sinking particles; unlike NO_3_^–^ and PO_4_^3–^, there is little build-up of regenerated dFe that can be entrained back into the euphotic zone in the following winter (20). Additional comparisons with other GEOTRACES transects across the North Pacific indicate that zonal gradients in dFe are minor in the upper 300 m of the water column (<0.1 nM per 1000 km; *SI Appendix*, Fig. S5), meaning that transport of coastal Fe sources by the Kuroshio Current and extension are negligible. We therefore conclude that the springtime increase in isotopically-light dFe in the NPTZ—observed in both 2017 and 2019—must derive from atmospheric sources.

### The Contribution of Anthropogenic Iron.

The light Fe isotope signature observed during spring is inconsistent with natural aeolian sources from desert dust or soils entrained by wildfire, which should yield δ^56^Fe close to crustal values (+0.09 ‰) (3, 21, 22). In contrast, Kurisu et al. (4, 23, 24) have determined that soluble Fe in aerosols from East Asia is strongly fractionated, with an anthropogenic endmember δ^56^Fe of approximately –3.9 to –4.7 ‰. A recent report of δ^56^Fe in aerosols from the East China Sea also identified a similar endmember of –4.5 ‰ (25). The extremely light composition of *Fe_anthro_* provides an explanation for the low δ^56^Fe measured in soluble and bulk aerosols collected in both the Atlantic and Pacific (3–5, 21), as well as surface seawater in the NPTZ (11), and enables the factional contribution of *Fe_anthro_* to be calculated by two-component endmember mixing equations. However, the applicability of simple mixing models in seawater is hindered by ecosystem processes that also modify δ^56^Fe, including phytoplankton uptake and complexation with organic ligands (14, 26, 27), which lead to a baseline δ^56^Fe that exceeds crustal values at the renewing of the seasonal cycle. Biological fractionation will also increase seawater δ^56^Fe during the spring bloom period, when phytoplankton Fe demand and aeolian Fe supply are greatest. Thus, ecosystem fractionation over multiple timescales serves to underestimate *Fe_anthro_* calculated from standard two-component mixing models.

To improve estimates of the anthropogenic Fe contribution to surface waters, we leveraged the distinct trajectories of isotope mixing and biological fractionation in concentration-isotope space to separately constrain Fe sources and sinks in the surface mixed layer (Fig. 2*F*, see *SI Appendix*). A stable seasonal Fe cycle requires balance between these two processes—a constraint that forces two intersections of a hyperbolic mixing curve (dFe sources) with a linear trend describing open-system fractionation (dFe sinks) (*SI Appendix*, Fig. S6). The resulting cycle describes our measurements with an ecosystem fractionation factor of 0.998 ± 0.001 (1 SD), which overlaps with previous estimates (27), and a soluble aerosol supply endmember of –1.90 ± 0.40 ‰ (1 SD). This latter value represents the integrated flux of dFe from atmospheric deposition and assumes a similar seasonality in transport of natural and anthropogenic aerosols across the Pacific, which is reasonably consistent with available observations (28, 29). The derived –1.90 ‰ atmospheric δ^56^Fe value also falls within the reported range of Fe released from Asian, North American, and European aerosols during leaching experiments (−0.4 to −3.7 ‰) (3, 25, 30, 31). After applying endmembers for the natural (+0.09 ± 0.10 ‰) and anthropogenic (–4.3 ± 0.40 ‰) sources described above, this translates to a 45 ± 10 % contribution of *Fe_anthro_* to soluble atmospheric Fe supply, and a 39 ± 9 % contribution to mixed layer dFe sources overall (the latter estimate includes a contribution from winter mixing, see *SI Appendix*). The relative contribution of *Fe_anthro_* is within the range of recent estimates from atmospheric models for the North Pacific (1, 2, 10, 32, 33), and the inferred flux of soluble Fe from both natural and anthropogenic sources (17 μmol m^−2^ year^−1^) is similar to observations at the Hawaii Ocean Time-series in the North Pacific Subtropical Gyre, which also receives a substantial flux of Asian aerosols during spring [>10 μmol Fe m^−2^ year^−1^] (34). Relative to preindustrial times, our results imply that human activities have increased the supply of dissolved Fe to North Pacific surface waters by over 50 %.

### Evidence for Phytoplankton Fe Stress in Spring.

**Fig. 3.**
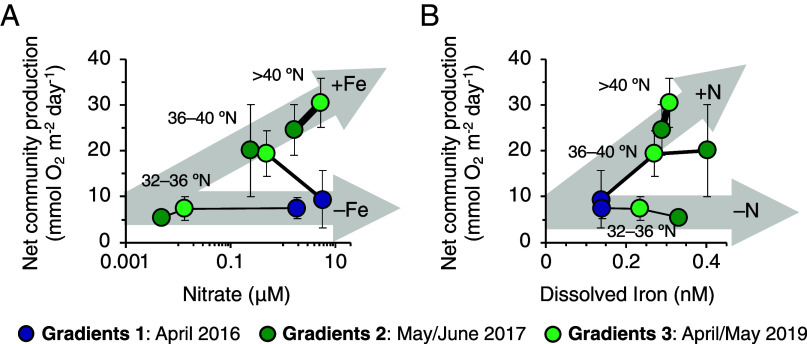
Evidence for Fe–N colimitation of net community production (NCP, in mmol O_2_ m^−2^ day^−1^) across the NPTZ during Spring. (*A*) When dFe is elevated (> 0.2 nM), NCP increases linearly with increasing NO_3_^–^ (gray arrow labeled “+Fe”). (*B*) When NO_3_^–^ is present at concentrations > 0.1 μM, NCP increases with increasing dFe (gray arrow labeled “+N”). NCP measurements at high resolution were grouped into 4 ° latitudinal bins and aligned with mean values for NO_3_^–^ and dFe (*SI Appendix*, Figs. S1 and S7). Significant effects of dFe, NO_3_^–^, and the interaction of dFe with NO_3_^–^ on NCP were confirmed with two-way ANOVA (*P* < 0.05, *SI Appendix*, Table S1).

Our construction of the Fe cycle shows that springtime supply of atmospheric Fe coincides with periods of high plankton productivity and nutrient uptake (Fig. 3). Farther north, in the high-NO_3_^–^ waters of the Subarctic Pacific, Fe deficiency is known to limit primary production and carbon export year-round (35, 36). Similar Fe limitation (or colimitation) has also been observed in transitional ecosystems in other ocean basins (37), suggesting that the progression of spring phytoplankton blooms in the NPTZ, and thus, the transition to oligotrophic conditions, depends on the arrival of this atmospheric Fe source. This conceptual model is consistent with evidence for Fe–N co-regulation of net community production in the NPTZ (Fig. 3; two-way ANOVA: *P* < 0.05; *SI Appendix*, Table S1). However, seasonal timing of the northward TZCF migration is also shared by increasing light, shoaling of the surface mixed layer, and seasonal warming (*SI Appendix*, Fig. S3): factors that can independently promote nutrient uptake by phytoplankton.

To investigate the potential for ecosystem Fe limitation in the NPTZ, we searched for known indicators of phytoplankton Fe stress in eukaryotic metatranscriptomes, collected at high spatial resolution on the Gradients expeditions during spring 2016, 2017, and 2019. At present, transcriptomic markers of Fe stress are best characterized in diatoms (Bacillariophyta) (38); prior experiments in the Subarctic Pacific (39) and in culture have confirmed that chronic Fe stress in natural diatom populations leads to predictable upregulation of genes encoding flavodoxin (*fld*) (40) and proteorhodopsin (*PR*) (41), which reduce the Fe demand of photosynthesis, as well as the “iron starvation induced proteins” *ISIP1* (42) and *ISIP2a* (43), which enhance Fe acquisition.

**Fig. 4.**
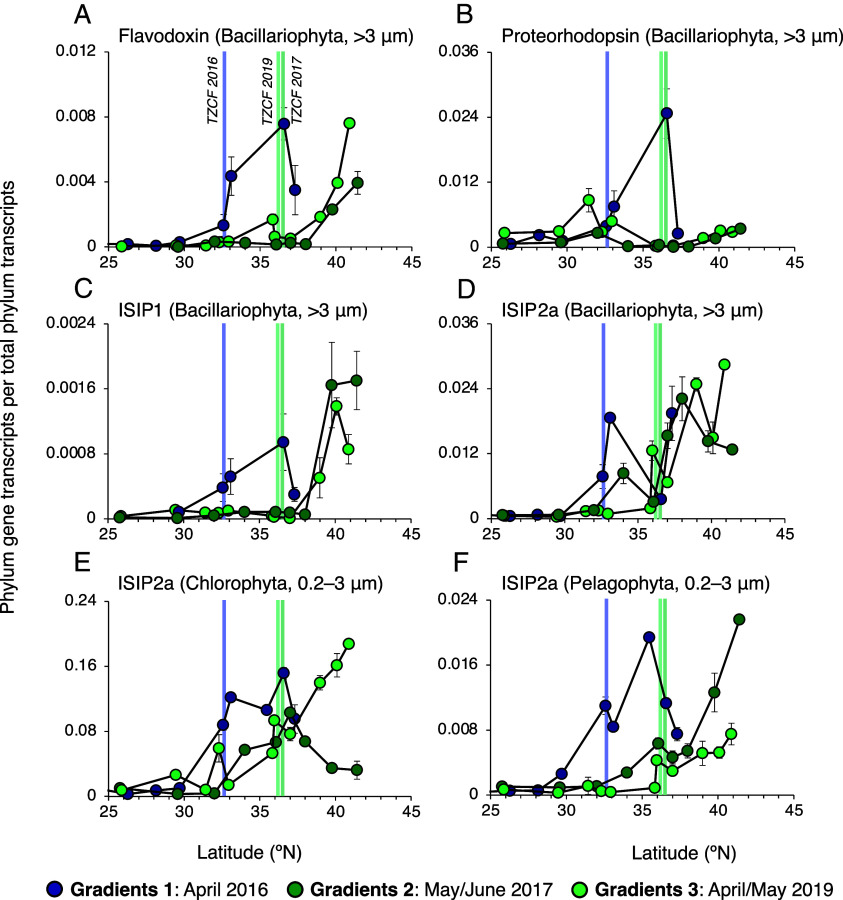
Relative expression of iron-stress related genes from metatranscriptomes collected on the Gradients cruises (April 2016, June 2017, April 2019). Phylum-normalized abundance of diatom transcripts for (*A*) flavodoxin clade 2, (*B*) proteorhodopsin, iron-stress-induced proteins (*C*) *ISIP1* and (*D*) *ISIP2a*. (*E* and *F*) Expression of *ISIP2a* in Chlorophyta and Pelagophyta, which are generally more abundant than diatoms (Bacillariophyta) in the NPTZ (note also that *ISIP2a* expression accounts for >10 % of all functionally annotated Chlorophyt transcripts). In all plots, vertical bars correspond to the location of the 0.2 mg m^−3^ TZCF, based on in situ measurements (18).

On all three spring expeditions, the expression of diatom Fe-stress markers increased north of the TZCF (Fig. 4). In April 2016, low dFe and δ^56^Fe < 0 ‰ suggest that sampling occurred near the onset of atmospheric deposition (consistent with expectations from isotope mixing, see calculation in *SI Appendix*). Low rates of net community production were also observed on both sides of the TZCF (*SI Appendix*, Fig. S7). During this expedition, expression of all four Fe-stress genes increased north of 32 °N, matching both the latitude of the TZCF and the transition to a high NO_3_^–^ and PO_4_^3–^ regime (Fig. 2 *B* and *C*). Meanwhile, increased expression of Fe-stress markers on the 2017 and 2019 cruises was not observed until ~37 °N, which also corresponded with the TZCF. Increased dFe in 2017 and 2019 (Fig. 2*D*) were accompanied by high rates of net community production north of the TZCF (*SI Appendix*, Fig. S7) (18), which could not be supported solely from the Fe supplied by winter mixing (*SI Appendix*). Continued expression of diatom *fld*, *ISIP1,* and *ISIP2a*, despite increased dFe, suggests that diatoms were still subject to Fe stress north of the TZCF in 2017 and 2019 and that productivity would continue to increase with additional Fe input. Similar expression of *ISIP2a*, which encodes the widely distributed Fe transporter phytotransferrin (43), was also observed in chlorophytes and pelagophytes (Fig. 4 *E* and *F*)—taxa that are less studied in culture but were more abundant than diatoms in the NPTZ (18).

The metatranscriptomic dataset indicates that the NPTZ is a seasonally Fe-stressed biome and that the TZCF can serve as an approximate southern boundary of an Fe-stressed regime that continues northward into the permanently Fe-limited subarctic Pacific. Indeed, correlations between increased expression of Fe-stress markers and the concentration ratio of NO_3_^–^:dFe were observed across the three springtime cruises (*SI Appendix*, Fig. S8). To the extent that light is sufficient for photosynthesis (44) and the physiological Fe stress described by these marker genes reflects Fe-limitation of phytoplankton growth rates, our results imply that any supplemental Fe supply north of the TZCF will increase productivity (Fig. 3), at least until macronutrients are exhausted and the ecosystem reverts to nitrogen limitation.

### Satellite Observations of Ecosystem Change in the NPTZ.

**Fig. 5.**
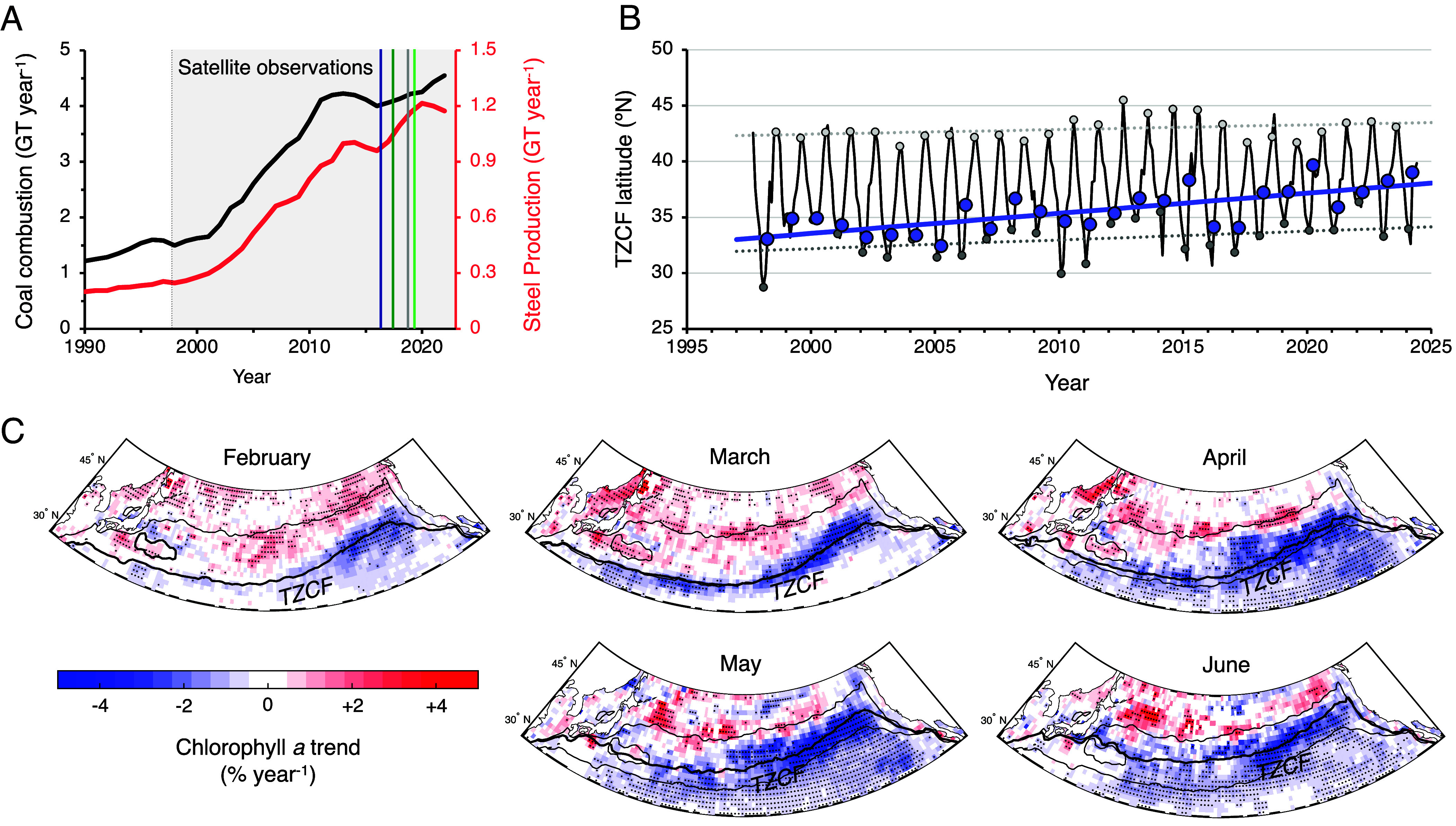
Evidence for ecosystem shifts in the NPTZ during the recent satellite era. (*A*) Coal combustion (black line) and steel production (red line) from East Asia (China, Japan, South Korea, and Taiwan) in GT year^−1^, based on reconstructions from the Global Carbon Project (45) and industry statistics from the World Steel Association (46). (*B*) Time series of the mean TZCF latitude between 140 to 180 °W, based on the OC-CCI multisensor merged chlorophyll *a* product (47). Blue circles mark the TZCF in April, which is shifting northward at a rate of 1.81 °N per decade (bolded blue line, *P* < 0.05). Dark and light gray circles respectively indicate the seasonal minimum (February) and maximum (August) in the TZCF, with dotted lines showing weaker trends for these months (*SI Appendix*, Table S2). (*C*) Monthly trends in satellite-detected chlorophyll *a* within 1° × 1° bins for the 1998 to 2024 period, normalized to monthly mean chlorophyll *a* (see *SI Appendix*, Fig. S7 for zonally integrated regressions). The thick black line in each panel marks the mean position of the TZCF for each month, while thin black lines mark the February and August TZCF in all panels. Black dots indicate statistically significant trends. Note the trend for increasing chlorophyll *a* in waters north of the TZCF and decreasing trend at the TZCF. See also *SI Appendix*, Fig. S9.

In the North Pacific, *Fe_anthro_* perturbation has been rapid and recent (10). While the relative contribution of steel production and coal combustion to *Fe_anthro_* emissions is debated (32, 33), both processes now occur annually in East Asia at the gigaton scale, increasing >threerfold since 1997, a year that marks the beginning of global and contiguous satellite ocean color observations (Fig. 5*A*).

We searched for evidence of *Fe_anthro_*-driven impacts on phytoplankton populations in the broader NPTZ region over this time period using monthly-resolved satellite products of chlorophyll *a* concentration produced by the Ocean Color Climate Change Initiative (OC-CCI) (47), which integrates observations across multiple ocean color platforms to form a continuous 26 y record. Between 1998 and 2024, the TZCF latitude has shifted northward during spring (Fig. 5*B*), increasing at a maximum rate of 1.81 ± 0.33 °N per decade in April (R^2^ = 0.54, *P < 0.05*). A similar northward migration is observed in other satellite chlorophyll *a* products, which apply different methods for sensor calibration and merging (*SI Appendix*, Fig. S9), and from satellite particulate organic carbon, derived from quasi-independent observations of particle backscattering (*SI Appendix*, Fig. S10). In this region, the OC-CCI chlorophyll *a* record is long enough to separate secular trends in the TZCF from internal variability forced by the Pacific Decadal Oscillation and the North Pacific Gyre Oscillation, both of which correlate with monthly TZCF anomalies, but with residuals that retain a significant linear trend during April and May (1.25 °N per decade in April, R^2^ = 0.39, *P < 0.05*; see *SI Appendix* and *SI Appendix*, Fig. S11 and Table S2). In the context of the seasonal cycle, the >3 °N shift in the April TZCF implies that the recent ecological condition of the NPTZ in April (38.0 ± 1.5 °N for 2020 to 2024) is now similar to its June position at the beginning of the century (37.6 ± 0.9 °N for 1998 to 2002).

We propose that the faster springtime retreat of the TZCF is, in part, a consequence of the large increase in the flux of *Fe_anthro_* to the Pacific Ocean since 1997. A physiological, rather than physical, driver of the spring TZCF trend is suggested by the bimodal trends in chlorophyll *a* observed in spring (Fig. 5*C*). In waters near the TZCF, chlorophyll *a* has decreased over the last 26 y, while higher biomass waters north of the TZCF mostly show an increasing trend until June (Fig. 5*C* and *SI Appendix*, Figs. S9, S12 and S13). Increasing chlorophyll *a* in NO_3_^–^ rich waters north of the TZCF is consistent with lessened Fe stress over the 1998 to 2024 period. In turn, more rapid NONO_3_^–^ depletion—enabled by an increased Fe supply—would lead to an earlier onset of nitrogen limitation, resulting in the decreasing trend in chlorophyll *a* at the TZCF.

It must be emphasized that the concentration of phytoplankton chlorophyll *a* is sensitive to several environmental factors and that long-term trends cannot be unambiguously attributed to anthropogenic Fe input. On a cellular level, chlorophyll *a* will increase in response to increases in temperature or decreases in light, in addition to relief of nutrient stress (48, 49). Alternatively, reductions in vertical mixing or southward currents (which would decrease NO_3_^–^ supply) would be expected to reduce phytoplankton biomass and chlorophyll *a* in the surface mixed layer. We examined potential changes to temperature, photosynthetically active radiation, surface currents, and mixed layer depth using a combination of satellite and in situ databases (*SI Appendix*, Fig. S10). Based on the temperature sensitivity of cultured phytoplankton (48), the current magnitude of regional warming (~0.5 °C decade^−1^) is not expected to yield a large increase in cellular chlorophyll. There also appears to have been no change in surface irradiance (<1% change over 26 y across the NPTZ). Finally, we were unable to identify statistically significant trends in mixed layer depth in the NPTZ using the Argo Mixed Layer database (50) or meridional current velocities, based on the OSCAR reanalysis product (see *SI Appendix* and *SI Appendix*, Fig. S14). Furthermore, multiple linear regressions that accounted for interannual variations in both processes did not explain the northward shift in the April TZCF (*SI Appendix*, Figs. S15 and S16).

While declines in mixed layer depth are anticipated as the ocean continues to warm (51), decreasing NO_3_^–^ input due to shallower winter mixing would not explain the increasing trend in chlorophyll *a* between 40 to 44 °N (Fig. 5*C* and *SI Appendix*, Fig. S12). This trend is observed in multiple merged chlorophyll *a* products (*SI Appendix*, Fig. S13) and, presumably, requires an increasing supply of a limiting nutrient (*SI Appendix*, Fig. S17). Indeed, the increase in chlorophyll *a* can be supported by the estimated magnitude of *Fe_anthro_* (*SI Appendix*). We also note that similar bimodal changes in springtime chlorophyll *a* in response to *Fe_anthro_* addition have also been simulated by the PISCES biogeochemical model (which accounts for nutrient and nonnutrient controls on phytoplankton chlorophyll *a*) (14), along with a northward retreat of the modeled chlorophyll front and a decrease in surface NO_3_^–^ (*SI Appendix*, Fig. S18), as argued here based on observations.

Our synthesis of shipboard and satellite observations provides an empirical basis for asserting the presence of, sensitivity to, and ongoing impacts from *Fe_anthro_* emissions to an important open ocean ecosystem. These conclusions support predictions from biogeochemical models, which have described potential shifts in plankton community composition, productivity, and nutrient limitation near the NPTZ region as a consequence of sustained input of *Fe_anthro_* (7, 14). Separate simulations with similar models have emphasized that anthropogenic CO_2_ emissions will increase the stratification of the water column over the 21st century, decreasing macronutrient supply from the subsurface and driving a poleward expansion of oligotrophic provinces (51, 52). We have shown that regional Fe supply is dominated by atmospheric sources and is relatively insensitive to stratification. Therefore, we posit that the effect of *Fe_anthro_* on temperate, seasonally Fe-stressed regions like the NPTZ is synergistic with the anticipated impacts from ocean warming and stratification, combining to result in a faster retreat of ecosystem boundaries like the TZCF over the 21st century. Similarly, *Fe_anthro_* emissions during industrialization of North America and Europe, as well as extensive transatlantic coal-powered shipping in the early 20th century, may have perturbed the biogeochemistry of other ocean basins, especially the North Atlantic.

## Materials and Methods

### Oceanographic Expeditions and Methods for Dissolved Iron and δ^56^Fe.

The three springtime cruises along 158 °W were conducted as part of the Simons Collaboration on Ocean Processes and Ecology program: Gradients 1 (*R/V Ka’imikai-O-Kanaloa*, April 19 – May 4, 2016), Gradients 2 (*R/V Marcus G. Langseth*, May 25 – June 13, 2017), and Gradients 3 (*R/V Kilo Moana*, April 9–30, 2019). Key biogeochemical descriptions of the Gradients cruises are described in previous publications (11, 18). The GEOTRACES GP15 cruise primarily sampled along 152 °W (*R/V Thomas G. Thompson*, Sept 18–October 21, 2018); descriptions of the GP15 dFe and δ^56^Fe datasets are available in refs. 53 and 54. Trace metal clean sampling procedures were used for all cruises. Samples for dFe were acidified and extracted with Nobias PA-1 resin, with additional purification prior to measurement of δ^56^Fe by anion exchange chromatography following Conway et al. (55) (see *SI Appendix* for full description). All individual dFe and δ^56^Fe measurements used in this study can be found in Dataset S1. Nitrate and phosphate concentration data for all expeditions are publicly available (56–59).

### Iron-Stress Indicators in Metatranscriptomes.

Assembled and quality-controlled environmental metatranscriptome sequences derived from the Gradients cruises (60, 61) were queried for homology to known Fe-stress induced genes using hmmsearch (HMMER version 3.1b2; parameters: -E 0.00001) (62). Custom-made hmm-profiles, generated using reference protein sequences described in the literature for *ISIP1* and *ISIP2a* (63) and flavodoxin-clade 2 (40), were aligned with Multiple Alignment using Fast Fourier Transform (MAFFT) version 7.313 (parameters: – localpair–maxiterate 100–reorder–leavegappyregion) (64) and masked at positions with 25% or more gaps. For rhodopsin, the hmm profile for bacteriorhodopsin-like proteins (Pfam: PF01036) was used. Environmental hits were further selected by RAxML Evolutionary Placement Algorithm (-m PROTGAMMAILG) (65) analysis onto their respective RAxML-generated reference trees (66). Flavodoxin reference sequences were retrieved from ref. 40; rhodopsin reference sequences were retrieved from ref. 67; *ISIP2* reference sequences were retrieved from ref. 63. Only environmental sequences mapping to clade-2 for flavodoxin, *ISIP2a* for *ISIP2*, and to proteorhodopsin clade D were included in the analysis. *ISIP1* environmental sequences were selected based on homology by MAFFT alignment against the small diatom-specific *ISIP1* gene family. Transcript abundance of the selected environmental sequences was obtained from kallisto quantification (68) against the assembled metatranscriptomes. The relative transcript abundance of each gene was calculated against the total transcript abundance within the functional-annotated and assembled transcript pool at the phylum level.

### Satellite Products and Other Contextual Datasets.

Monthly OC-CCI chlorophyll *a* datasets (4 km, version 6, 9/1997–6/2024) were downloaded from https://www.oceancolour.org/ (69). The OC-CCI chlorophyll *a* combines level-1 reflectance data for several sensors (MODIS-AQUA, VIIRS-NPP, OLCI-A, and MERIS) and level-2 data from SeaWiFS prior to deriving chlorophyll *a* concentration (47). To identify the TZCF, data were averaged zonally between 140 to 180 °W (null-values excluded) and a six point (~0.25 °) latitudinal moving average was applied. The TZCF was defined as the southernmost latitude between 25 to 46 °N where chlorophyll *a* exceeded 0.2 mg m^−3^. All data processing was performed in MATLAB R2022 with linear regressions conducted using the *fitlm* function. Linear regressions of monthly anomalies were performed for the entire timeseries (*SI Appendix*, Table S2, “All”) and subset by month. Trends were identified as significant if *P < 0.05* (*SI Appendix*, Table S2). Trends in coarse-binned (1° × 1°) data were also calculated across the entire NPTZ, also using the *fitlm* function.

Motivated by recent concerns regarding the robustness of individual merged chlorophyll *a* datasets (70), additional merged products were examined to confirm that the trends observed in OC-CCI were robust (*SI Appendix*, Figs. S7 and S10). These included Yu et al. (2023) chlorophyll *a* product (71): v1.0 accessed from https://doi.org/10.5281/zenodo.7092220 (9/1997–12/2020), the Globcolour dataset distributed by CMEMS at: https://data.marine.copernicus.eu/products (4 km, 9/1997–7/2024, product ID: OCEANCOLOUR_GLO_BGC_L4_MY_009_104), and two Globcolour datasets distributed by Hermes (https://hermes.acri.fr/) for Type 1 hlorophyll *a* [CHL1, L3m, 25 km, weight-averaged “AVW”] (72) and model-derived “GSM” (73) (9/1997–4/2023). Globcolour AVW datasets for particulate organic carbon (74), aerosol optical thickness at 550 nm [T550] (75), and photosynthetically active radiation (76) were also investigated (*SI Appendix*, Figs. S3 and S8). Sea surface temperature data (SST4, 9 km) were downloaded from NASA Ocean Color (https://oceancolor.gsfc.nasa.gov/) for the MODIS-AQUA sensor between 2002 and 2023.

Additional datasets utilized in this work include the Argo mixed layer properties atlas (50), intercalibrated dFe datasets in the GEOTRACES Intermediate Data Product 2021 (77), country-specific coal combustion statistics from the Global Carbon Budget (45) (converted from CO_2_ units following the EPA Greenhouse Gases Equivalencies Calculator: https://epa.gov/energy/greenhouse-gases-equivalencies-calculator-calculations-and-references#), and publicly available data of national steel production compiled from the World Steel Association (46). When possible, these figures were checked against peer-reviewed compilations (78) and USGS Mineral Commodity Summaries and Minerals Yearbooks for Iron and Steel (https://www.usgs.gov/centers/national-minerals-information-center/iron-and-steel-statistics-and-information) and generally agreed to within a few percent. In Fig. 1, uniform colormaps originated from ref. 79.

## Supplementary Material

Appendix 01 (PDF)

Dataset S01 (XLSX)

## Data Availability

1) Oceanographic Bottle data (Zenodo & BCO-DMO DOI’s + Dataset S1) 2) Metatranscriptome data (Zenodo DOI: https://doi.org/10.5281/zenodo.12630398) (61) 3) Satellite data: publicly availably from oceancolour.org (69). Data have been deposited in Zenodo and BCO-DMO (https://doi.org/10.5281/zenodo.3762278, https://doi.org/10.5281/zenodo.3601594, https://doi.org/10.5281/zenodo.7782668, https://doi.org/10.26008/1912/bco-dmo.777951.6, and http://doi.org/10.26008/1912/bco-dmo.883862.1) (54, 56–59).
