# Supplementary material for: Anthropogenic iron alters the spring phytoplankton bloom in the North Pacific transition zone
Source: Proc Natl Acad Sci U S A. 2025 Jun 2;122(23):e2418201122. doi: 10.1073/pnas.2418201122 (PMC12168011; doi:10.1073/pnas.2418201122)
Supplement: Supplementary file 1 — Appendix 01 (PDF) [file pnas.2418201122.sapp.pdf]

# Supporting Information for
Anthropogenic iron alters the spring phytoplankton bloom in the North Pacific Transition Zone

Nicholas J. Hawco, Tim M. Conway, Sacha N. Coesel, Benedetto Barone, Emily A. Seelen, Shun-Chung Yang, Randelle M. Bundy, Paulina Pinedo-Gonzalez, Xiaopeng Bian, Matthias Sieber, Nathan T. Lanning, Jessica N. Fitzsimmons, Rhea K. Foreman, Daniela König, Mora J. Groussman, James G. Allen, Lauren W. Juranek, Angelicque E. White, David M. Karl, E. Virginia Armbrust and Seth G. John

Nicholas Hawco
Email: hawco@hawaii.edu

**This PDF file includes:**

Supporting text
Figures S1 to S18
Tables S1 to S2
Legends for Dataset S1
SI References

**Other supporting materials for this manuscript include the following:**

Datasets S1

## Supporting Information

### Sampling and analysis of dissolved iron (dFe) and isotopic composition ($\delta^{56}Fe$)

For the Gradients cruises, water samples for dissolved iron (dFe) and iron isotopic composition ($\delta^{56}Fe$) were collected following GEOTRACES 'cookbook' protocols using an underway tow-fish and 'trace metal clean' rosette system, with samples filtered through a 0.2 μm capsule filter and acidified to a pH of 1.8 for several months prior to analysis. dFe was measured by isotope dilution after pre-concentration with a SeaFAST S2 Pico preconcentration system (Elemental Scientific) and analyzed by inductively coupled plasma mass spectrometry (ICP-MS) with an Element 2 instrument (Thermo Scientific) at the University of Southern California. These samples were analyzed contemporaneously with samples reported in other publications (1–3), which describe acceptable comparisons from GEOTRACES consensus standards.

For $\delta^{56}Fe$ analysis, acidified samples were amended with a $^{57}Fe$, $^{58}Fe$ double spike, then extracted, purified, and analyzed following the procedures described by Ref. (4). All Fe isotope samples were measured by multi-collector ICP-MS instruments (Thermo Scientific Neptune) located at either the University of South Carolina (Gradients 1 and 2 samples (13)) or the University of South Florida (Gradients 3). dFe and $\delta^{56}Fe$ data and methods from the Gradients 2 cruise have been published previously (1), and identical methods were used for Gradients 1 and 3 samples reported here. Long-term accuracy and reproducibility for $\delta^{56}Fe$ measurements corresponding to the Gradients $\delta^{56}Fe$ data is estimated by repeated analyses of the NIST SRM 3126a secondary standard from September 2021 to April 2024 across three Thermo Scientific Neptune instruments at the University of Southern California, University of South Florida, and Academia Sinica is +0.35 ± 0.07 (2SD, n = 111).

For GEOTRACES GP15, surface tow-fish and trace metal rosette water sampling and filtration (0.4 μm pore size), and acidification procedures are described previously (5–7). GP15 samples were processed and measured for dFe and $\delta^{56}Fe$ analysis following previously published methods using Nobias PA-1 extraction and the Fe double spike technique with analysis by Thermo Scientific Neptune ICP-MS at the University of South Florida (4, 8, 9), analogous to the procedures used for the Gradients samples. dFe concentrations were calculated using the isotope dilution technique based on the $^{57}Fe/^{56}Fe$ ratios from the isotope analyses and are assumed to have an uncertainty of 2% (4). The long-term reproducibility for $\delta^{56}Fe$ at USF (regarded as a conservative estimate of uncertainty) is similarly estimated from repeated analyses of NIST-3126, giving $\delta^{56}Fe$ = +0.36 ± 0.05 ‰ (2SD, n = 524 during 47 sessions), which agrees with values from USC, and from other studies (9). We apply 0.05 ‰ for GP15 samples and 0.07 ‰ for the Gradients samples as an estimate of 2σ uncertainty on $\delta^{56}Fe$, except for low concentration samples where the larger internal error is considered a more conservative estimate of uncertainty.

To determine if measurements were significantly different across the NPTZ, we conducted 1-way ANOVAs with post-hoc Tukey tests in MATLAB (*anova1* and

*multicompare* commands) on dFe and $\delta^{56}$Fe datasets, using $p = 0.05$ as a significance threshold.

Datasets for dissolved phosphate and nitrate concentrations from the Gradients cruises used in this work can be accessed on Zenodo (zenodo.org/records/3762278, zenodo.org/records/3601594, zenodo.org/records/7782668). Data from published net community production measurements (10) are available at 10.5281/zenodo.3958315, 10.5281/zenodo.4009653, 10.5281/zenodo.4079505, and 10.5281/zenodo.4104636. Nutrient data from GP15 used in this study can be accessed via the Biological and Chemical Oceanography Data Management Office website (bco-dmo.org/dataset/77795).

**Estimating Anthropogenic Contributions**

The overlapping influence of endmember mixing and open-system biological fraction in our surface ocean dataset precluded the application of a single equation to describe all dFe and $\delta^{56}$Fe observations. Open-system biological fractionation (following a Rayleigh fractionation factor $\alpha_{bio}$), according to the equation:

$$\delta^{56}Fe_i = \delta^{56}Fe_{initial} + 1000\left(1 - \frac{1}{\alpha_{bio}}\right)\left(1 - \frac{dFe_i}{dFe_{initial}}\right) \quad (1)$$

should result in a linear relationship between dFe concentration and $\delta^{56}$Fe. At present, the specific processes that result in dFe fractionation are poorly defined, and $\delta^{56}$Fe observations may reflect the net effect of multiple reactions (uptake, regeneration, reduction, complexation, adsorption, etc.) that contribute to an apparent 'ecosystem' fractionation effect ($\alpha_{bio}$) describable by Eq. 1. An open system approach is justified by the co-occurrence of dust input and high biological productivity observed in spring (see Fig. 1, 2a).

Endmember mixing follows an inverse relationship, according to the equation:

$$\delta^{56}Fe_i = \frac{1}{dFe_i}\left(dFe_{initial} \times \delta^{56}Fe_{initial} + \delta^{56}Fe_{source} \times (dFe_i - dFe_{initial})\right) \quad (2)$$

and will appear as a negative excursion from a linear trend. In a closed cycle, the mixing equation intersects with the fractionation relationship at both the seasonal minimum and maximum dFe. Thus, the result of Eq. 1 can be used to define $dFe_{initial}$ and $\delta^{56}Fe_{initial}$ for Eq. 2, and vice versa.

We implemented a fitting routine to objectively sort observations into 'fractionation-dominated' and 'mixing-dominated' groups. An initial, Type-2 linear regression was performed to latitudinally-binned data in concentration-isotope space to create a ranking of residuals (Fig. S5a). Ranked negative residuals were progressively removed from the linear regression and assigned to a non-linear fit of Eq. 2, with one endmember representing the seawater baseline – defined by the solution to the linear regression at a specified (later, optimized) value of dFe, and the other endmember representing the $\delta^{56}$Fe of the atmospheric source. A combined $R^2$ for these two equations was calculated as:

$$R^2 = 1 - \frac{(ss_{fractionation} + ss_{mixing})}{ss_{tot}} \quad (3)$$

where $ss_{fractionation}$ and $ss_{mixing}$ is the sum of squares between observations and the fit of the fractionation-dominated or mixing-dominated datasets, respectively, and $ss_{tot}$ is the total variance. The maximum in the combined $R^2$, reflecting the total variance explained by both equations, was used to identify the optimal number of 'mixing-dominated' observations (n = 4 for the latitudinal-binned samples; n = 11 for individual observations) and the resulting best estimate of $\delta^{56}Fe_{source}$ (Fig. S5b-f). A range of $dFe_{initial}$ concentrations were applied (0.01 to 0.20 nM Fe) with the optimal fit (highest combined $R^2$) resulting from 0.09 nM (Fig. S5e). The derived endmember composition (–1.90 ± 0.40 ‰), reflecting the integrated aerosol supply endmember of soluble Fe, was then translated into a fractional contribution by applying a standard two-component mixing equation, using natural and anthropogenic $\delta^{56}Fe$ endmembers of +0.09 ± 0.10 and –4.3 ± 0.4 ‰, respectively. The later estimate is derived from the midpoint of the estimated range of industrial $\delta^{56}Fe$ endmember of –3.9 to –4.7 ‰, described by Kurisu et al. (11–13). Uncertainty at 1 SD was determined based on the propagated uncertainty of the best fit composition of the aeolian supply, and the above uncertainties of the natural and anthropogenic endmembers. The soluble Fe flux from the atmosphere was estimated assuming a spring/summer mixed layer depth of 30 m and the seasonal amplitude in dFe, defined by the intersections of the mixing and fractionation curves at 0.09 and 0.66 nM dFe, according to the best fit line in Fig. 2. A mixed-layer Fe residence time of 5 months can be inferred from this calculation, which is consistent with other characterizations of the Fe cycle in the North Pacific (14, 15).

**Analysis of satellite chlorophyll datasets**

To investigate if the significant long-term trends could be alternatively explained by known regional climate oscillations, linear regressions were conducted between the TZCF latitude and lagged NPGO and PDO indices. The index for PDO was downloaded from NOAA NCEI (ncei.noaa.gov/access/monitoring/pdo/) and the updated index for the NPGO from Ref. (16) was downloaded from o3d.org/npgo/npgo.php. Timeseries cross-correlation was used to identify the optimal lag between the PDO/NPGO and the TZCF anomaly, performed using the MATLAB *xcorr* function and were found to be 1 month for both indices (Table S2). Regressions of the TZCF anomaly and a) the PDO, b) NPGO, and c) their combination (form: $y = m_1*PDO + m_2*NPGO + b$) all returned significant correlations, with $R^2$ values of 0.21, 0.09, and 0.32, respectively (Table S2).

Residuals from the PDO, NPGO, and NPGO+PDO regressions were used to determine whether long-term linear trends in the TZCF latitude were robust. In each case, the linear trend for the entire timeseries remained statistically significant, but with a 4-fold reduction in trend magnitude in the combined case. For regressions specified by month, 6 of 12 months retained a significant trend after correction for the effects of the PDO, but only 2 months (April, May) in either the NPGO- or NPGO+PDO-corrected cases. Considering the NPGO+PDO-corrected case as the most conservative approach, northward TZCF trends in April and May remained statistically significant with slopes that were 7- and 4-times larger than for the entire timeseries, respectively (Table S2). From this analysis, we conclude that the TZCF record is strongly influenced by the PDO and NPGO, but that these factors cannot explain the long-term northward migration of the TZCF in springtime.

**Investigating changes in mixed layer depth and surface currents as drivers of chlorophyll trends**

Prior studies have emphasized macronutrient supply as a dominant influence of the seasonal cycle in the NPTZ, especially the roles of deep winter mixing (entrainment) and southward transport of nitrate-bearing surface waters (which includes Ekman transport) (17–19). Assessments of winter mixed layer depth (MLD) were conducted using the Argo Mixed layer database (20), which contained >34,500 individual MLD determinations within the NPTZ region (140–180 °W, 32–45 °N), based on the MLD algorithm described by Holte and Talley (21). Individual MLD values were grouped into 3276 monthly 1° latitudinal bins for each year in the 2002 – 2022 dataset (mean coverage = 11 MLD determinations per bin) and the median value was calculated.

Several attempts were carried out to search for long-term trends in the resulting MLD timeseries, none of which yielded statistically significant results. These approaches included linear regressions versus time and **a**) the monthly anomaly for mean MLD, after averaging across 32–45 °N for all months ($p = 0.12$), **b**) the monthly anomaly for mean MLD across 32–45 °N for each month (Fig. S10g; $p = 0.09$ for February and greater for all other months except August and September, which showed a small deepening trend), **c**) the monthly anomaly for mean MLD for each 1° latitude bin for each month (Fig. S14), and **d**) the maximum monthly MLD for each year (calculated from the 32–45 °N mean, $p = 0.19$). These non-significant regressions often produced negative slopes during January–March, consistent with expectations for declining winter MLD in response to ocean warming. The only possible conclusion at this point, however, is that the observed MLD record is too short or sparse to identify trends among the extensive interannual variability in MLD across the NPTZ region.

Similar regressions were conducted for the meridional velocity using the OSCAR surface currents product, which assimilates observations of surface winds, and sea-surface height to generate meridional and zonal current velocities in the surface mixed layer from 1993–2020 (22, 23). Regressions equivalent to those calculated for MLD (a–d in the preceding paragraph) also did not meet significant criteria ($p > 0.05$, see Figs. S10i, S14).

The absence of detectable long-term trends does not necessarily mean that these physical processes do not impact the chlorophyll *a* concentration or the TZCF latitude in the NPTZ region. To account for these effects, we conducted a multiple linear regression for April chlorophyll *a* concentration (mean from 32–44 °N) and TZCF latitude as a function of both the annual maximum of MLD and the annual maximum of southward current velocity (calculated as the maximum value of the NPTZ monthly mean) for the 2002–2020 timespan where all datasets overlap (see Fig. S15, S16 for scatter plots and timeseries of variables and residuals). For the regression against April chlorophyll *a*, we found a significant relationship with the annual maximum in mixed layer depth ($p = 0.003$) but not with the annual maximum in surface currents ($p = 0.07$), such that deeper winter mixed layers drove positive anomalies in the mean chlorophyll *a* concentration across the NPTZ ($R^2 = 0.47$). However, for the April TZCF latitude, no significant relationship was observed for either MLD or Ekman transport ($p > 0.05$; $R^2 = 0.21$) and

the residual for this regression retained a significant, increasing relationship with time ($p = 0.002$; $R^2 = 0.45$), with a slope equal to 2.06 ± 0.55 °N decade$^{-1}$ (Fig. S16). Thus, the northward migration in the TZCF cannot be explained by changes in MLD or meridional currents, at least to the extent that these processes are represented by the datasets used here.

Together, these regressions emphasize the importance of nitrate supply by winter mixing in determining spring phytoplankton biomass, while the partitioning of this biomass north and south of the TZCF appears to be regulated by other ecosystem processes that vary independently from MLD or meridional current velocity (and also appear to be changing with time). As a result, the TZCF appears to be relatively insensitive to changes in nitrate supply. Inspection of 1° x 1° maps and zonal means of chlorophyll a shows increasing chlorophyll a north of the TZCF throughout the winter and spring seasons (Fig. S12, S17), leading to positive annual trends at latitudes >40 °N. This latter observation is suggestive of an increasing supply of a limiting or co-limiting nutrient. Although not definitive, this is consistent with evidence for co-regulation of this ecosystem by both nitrate and iron, and an increasing supply of $Fe_{anthro}$ over the past 30 years.

**Calculation showing that $\delta^{56}$Fe will be affected by anthropogenic Fe before dFe concentration**

Significance testing by 1-way ANOVA with Post-hoc Tukey tests indicated that dFe concentration data on the Gradients 1 transect in April 2016 was not significantly different than data from the GP15 expedition ($p > 0.05$). However, similar testing did indicate a significant difference in $\delta^{56}$Fe between these cruises ($p < 0.05$). This counterintuitive result is expected based on the sensitivity of $\delta^{56}$Fe to small amounts of anthropogenic Fe input, which we demonstrate below.

After artificially mixing the GP15 2018 results (dark grey circles in Fig. 2) and averaging across all latitudinal bins, we expect the resulting concentration of dFe in the mixed layer to be 0.128 nM, with $\delta^{56}$Fe = +0.40 ‰. The mean dFe for the Gradients 1 (April 2016) dataset was 0.166 nM. If this small increase (+0.038 nM) is attributed to aeolian sources with an endmember $\delta^{56}$Fe = –1.90 ‰ (the outcome of our regression scheme in Fig. 2f), then the $\delta^{56}$Fe of the two-component mixture is calculated to be –0.12 ‰. This is very close to the mean of the April 2016 $\delta^{56}$Fe dataset, –0.13 ± 0.25 ‰, demonstrating that a statistically insignificant change in dFe can yield a statistically significant change in the Fe isotope composition.

**Calculation showing that the winter dFe supply by entrainment cannot support Fe-replete phytoplankton growth during the spring bloom period.**

An approximate calculation can demonstrate the insufficiency of the wintertime Fe supply in accounting for observed ecosystem productivity and export of $NO_3^-$. In 2017 and 2019, the high NCP observed >36°N was ~24 mmol $O_2$ m$^{-2}$ day$^{-1}$, which is convertible to C units using a photosynthetic quotient of 1.4 mol $O_2$ : mol C (24). Under steady state, this net production of $O_2$ requires C export out of the mixed layer that is equal to 17 mmol C m$^{-2}$ day$^{-1}$.

Imaging flow cytometry indicated that centric diatoms (*Thalassiosira* sp.) and coccolithophores (*Emiliana* sp.) were abundant phytoplankton groups during the 2017 and 2019 expeditions (10). Sunda and Huntsman (1995) have shown that the transition of Fe-limited growth to Fe-replete growth for *Thalassiosira oceanica* and *Emiliana huxleyii* occurs at an internal Fe quota of ~ 10 μmol Fe: mol C (25). Because the Fe quota in these species will continue to increase with higher Fe availability, an Fe export rate of at least 0.17 μmol Fe $m^{-2}$ $day^{-1}$ is required.

Our fitting routine estimates that the annual entrainment flux of Fe is approximately 2.7 μmol Fe $m^{-2}$ (= 0.09 nM best fit 'background' dFe x 30 m mixed layer). Based on the above, one can infer that diatoms similar to *T. oceanica*, growing at the cusp of Fe limitation (i.e. a cell quota = 10 μmol Fe: mol C) , would deplete the annual allowance of dFe from entrainment within 16 days (= 2.7 / 0.17). Without additional supply of Fe from the atmosphere, the ecosystem would likely be subject to strong Fe limitation and require extraordinary recycling measures to decouple carbon export from Fe export, as observed in permanent HNLC regions (e.g. Rafter et al. 2018; Boyd et al. 2014).

In terms of nitrate units, assuming a C:N ratio of 6.5, the ~ 0.09 nM Fe supply inferred from winter mixing would be equal to a drawdown of 1.3 μM, a small fraction of the 5–8 μM $NO_3^-$ observed north of the TZCF in April 2016 or 2019.

Note that there is a great deal of uncertainty regarding the 'correct' Fe:C ratio to use, which can vary from close to 1 μmol:mol in iron-limited diatoms from the Southern Ocean with heavily modified photosynthetic architecture (26) to >100 μmol:mol in Fe-replete *Pseudonitzschia* capable of storing excess Fe in ferritin (27).

Furthermore, we can show that the anthropogenic Fe supply can sustain ecosystem productivity. Our mass balance estimations suggest that the atmospheric flux of dissolved iron is approximately 17 μmol $m^{-2}$ $year^{-1}$, with a 45% contribution of anthropogenic sources. Thus, the natural flux of dust-borne Fe is 9.35 μmol Fe $m^{-2}$ year. Based on the AOD climatology – as well as direct measurements of aerosol collections in the North Pacific (28) – dust-borne Fe primarily arrives over a period of 3 months during March, April, and May. During this ~90 day dusty season, the dFe flux is 0.10 μmol Fe $m^{-2}$ $day^{-1}$, similar (but seemingly less than) the 0.17 μmol Fe $m^{-2}$ $day^{-1}$ flux of Fe out of the euphotic zone.

Adding in the anthropogenic contribution, the combined Fe flux would be 0.19 μmol Fe $m^{-2}$ $day^{-1}$, matching (or in excess of) the expected Fe export. Thus, the natural Fe supply is probably insufficient to support the observed periods of high productivity (again, using an Fe quota representing a physiological state at the cusp of Fe limitation), while the total estimated Fe flux approaches a system closer to mass balance and would allow springtime accumulation of dFe, consistent with our observations.

**Calculation showing that the anthropogenic Fe supply is sufficient to account for the observed increase in chlorophyll, north of the TZCF**

To determine if the anthropogenic Fe supply can support a reasonable long-term increase in chlorophyll *a*, we conducted the following calculation. The estimated annual atmospheric Fe supply (17 µmol $m^{-2}$ $y^{-1}$) is scaled to a daily rate using a 90 day approximation of the March–May dusty season, and multiplied by the estimated 45% anthropogenic contribution. This gives an approximate daily flux of new anthropogenic Fe equal to 0.085 µmol $m^{-2}$ $day^{-1}$. Each Fe atom is likely recycled at least 5 times prior to export (15, 29), meaning that the $Fe_{anthro}$ supply can support a steady-state biological Fe uptake of 0.43 µmol $m^{-2}$ $day^{-1}$. If it is further assumed that: a) the mixed layer is 30 m deep, and b) that a typical phytoplankton during the spring bloom period grows at a rate of 0.69 $day^{-1}$ (= 1 doubling $day^{-1}$), we calculate an increase of 21 pM Fe within phytoplankton biomass as a result of the $Fe_{anthro}$ flux.

As above, we use the 10 µmol Fe: mol C as a useful and appropriate phytoplankton Fe quota and apply a Chlorophyll : C quota of ~0.3 mmol : mol from the same source (25). Considering the molecular weight of chlorophyll *a* (893.5 g/mol), 1 pmol of Fe could therefore support 26.8 ng of chlorophyll *a*. Thus, a 21 pM increase in biotic Fe could potentially yield an increase in chlorophyll *a* concentration by 0.56 µg chlorophyll $L^{-1}$, which could easily double the observed chlorophyll in the NPTZ (see also Fig. S17).

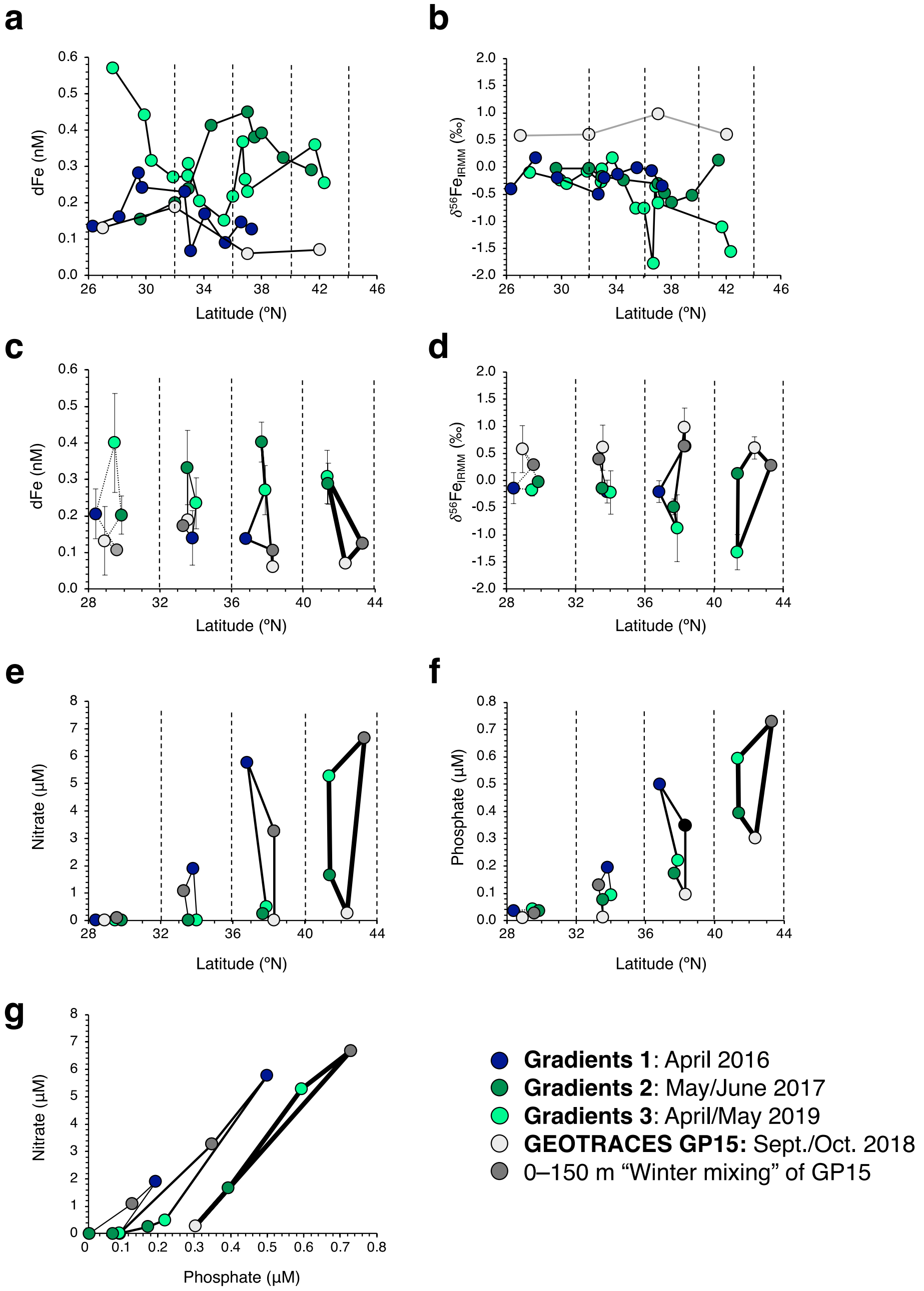


**Fig. S1.** Construction of a seasonal Fe cycle in the NPTZ by binning data from four oceanographic cruises. Latitudinal distribution of (**a**) dissolved iron (dFe) and (**b**) the isotopic composition of dFe ($\delta^{56}$Fe) in the near-surface ocean (0–50 m) along the Gradients 1, 2, and 3 transects and GEOTRACES GP15. When relevant, multiple samples from the same station were averaged. **c**) dFe and **d**) $\delta^{56}$Fe averaged within latitude bins (26–32, 32–36, 36–40, and 40–44 °N) for each cruise, with error bars showing the standard deviation of measurements within the bin. Impacts of winter mixing/entrainment from below the mixed layer (dark grey circles) are calculated as the 0–150 m integral of dFe based on the GP15 data ÷ 150 m to convert to a concentration; integrated values of $\delta^{56}$Fe are calculated analogously, but weighted by dFe concentration (see Fig. S2). Corresponding nitrate (**e**) and phosphate (**f**) distributions within the same latitudinal bins, and (**g**) their co-evolution throughout the seasonal cycle are shown for context. For each parameter in panels **a** – **f**, the mean latitude for each bin is plotted.

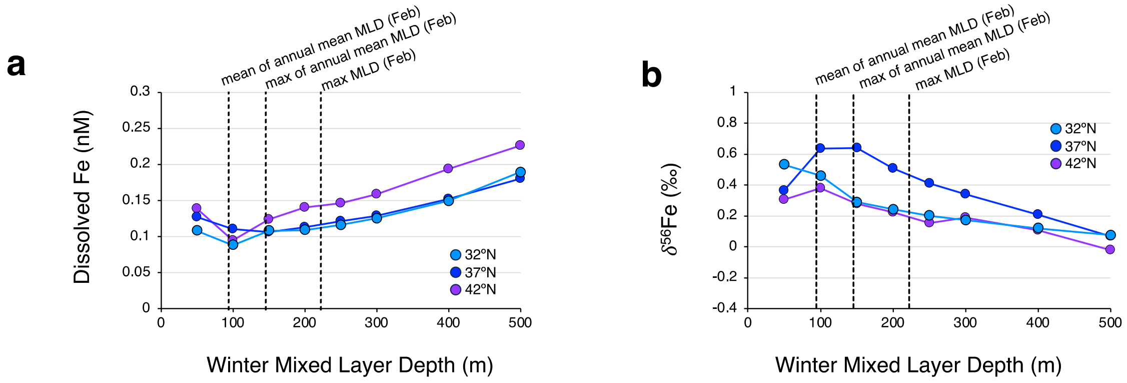


**Fig. S2.** Impact of winter mixed layer depth on entrainment of **a**) dFe and **b**) $\delta^{56}$Fe based on profiles at 4 stations across the NPTZ, sampled on the GP15 expedition. Values in (**a**) are calculated as the concentration if the water column is homogenized between the surface and depth of the winter mixed layer (i.e. the areal dFe inventory divided by the integration depth). Values in (**b**) are concentration-weighted to reflect isotope mixing. Vertical dashed lines indicate three statistics, based on the Argo float mixed layer atlas (20): mean February mixed layer depth between 2000–2022 in the NPTZ (32–44 °N, 140–180 °W), the maximum of the February mean between 2000–2022 , and the maximum observed mixed layer depth within a 1° × 1° grid. Annual maximum values for all 3 metrics occurred during February. Before processing, individual float profiles were grouped into a 1° × 1° × 1 month grid. If more than one profile occurred within the same grid point, the median was calculated and used for subsequent statistics.

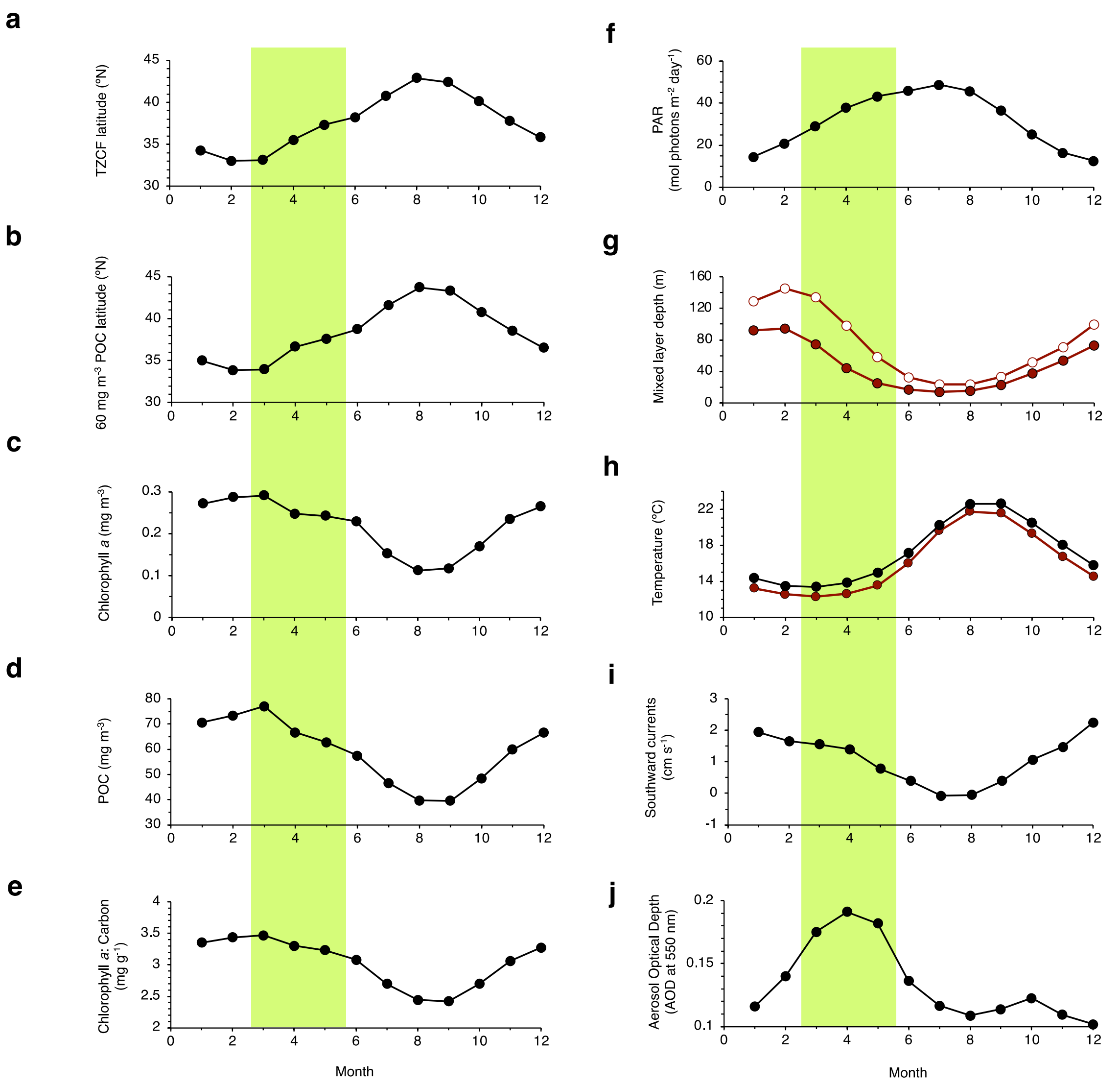


**Fig. S3.** Seasonal cycles of biological and environmental parameters in the NPTZ from satellite data and *in situ* observations (averaged between 32–44 °N and 140–180 °W). In all panels, green shading highlights the spring period between March and May. The springtime retreat of the Transition Zone Chlorophyll Front (**a**, TZCF) and an equivalent threshold of 60 mg carbon $m^{-3}$ derived from satellite-detected particulate organic carbon (**b**, POC) co-occurs with seasonal decreases in domain-averaged (**c**) chlorophyll *a*, (**d**) POC, (**e**) the chlorophyll *a*:C ratio, and increases in (**f**) photosynthetically active radiation (PAR). During this period, (**g**) the mixed layer depth decreases, (**h**) mixed layer temperature increases, (**i**) southward currents slacken, and (**j**) aerosol optical depth (AOD at 550 nm) is at its seasonal peak. Black circles in all figures represent satellite products, while red circles indicate Argo float data from 2002 – 2022 (20). All satellite data reflect multisensor merged products. Chlorophyll / TZCF: OC-CCI v6 between Sept 1997 to June 2024; POC, Chlorophyll *a*:C, PAR, and AOD: Globcolour L3m multi-sensor weight-averaged products (25 km) between Sept 1997 and April 2023; temperature: MODIS-AQUA SST4 (9 km), 2002 – 2023. The southward component of surface currents derives from monthly-averaged OSCAR surface velocity fields (22). Open circles in panel **g** reflects the latitudinal and monthly mean for an array of maximum observed values within 1° latitudinal bins, whereas closed circles reflect the latitudinal and monthly mean for an array of median values.

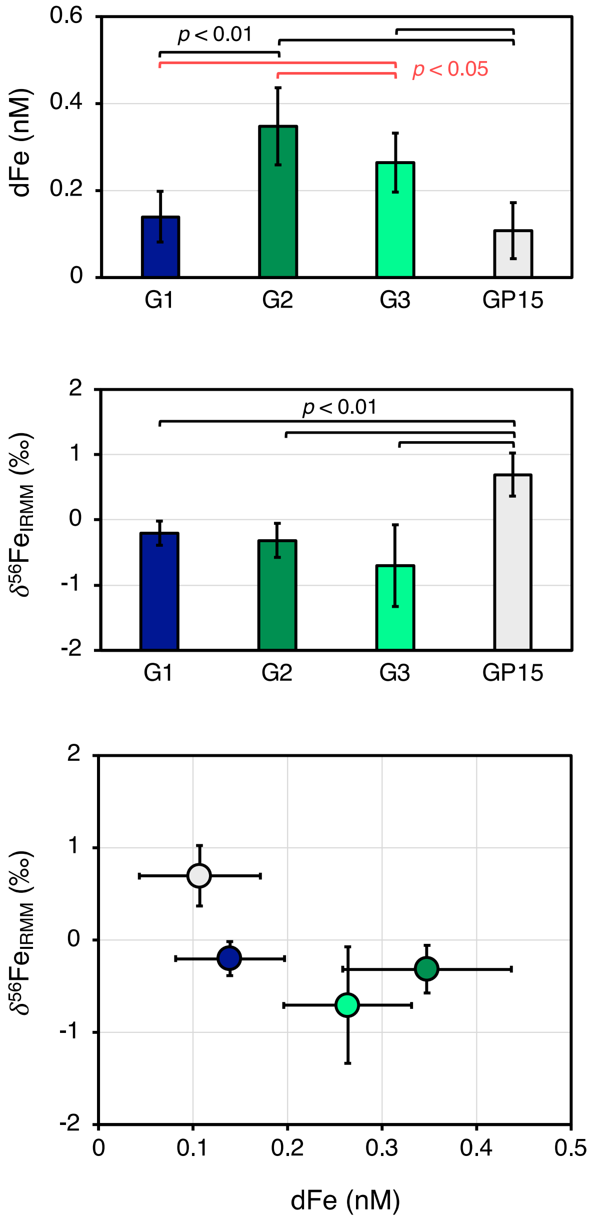


**Fig. S4**. Dissolved Fe and $\delta^{56}$Fe in the North Pacific Transition Zone (32–44 °N), grouped by expedition. Brackets show results of significance testing via 1-way ANOVA with post-hoc Tukey tests. Significant differences from pairwise comparisons at thresholds $p < 0.05$ and $p < 0.01$ are shown in red and black, respectively.

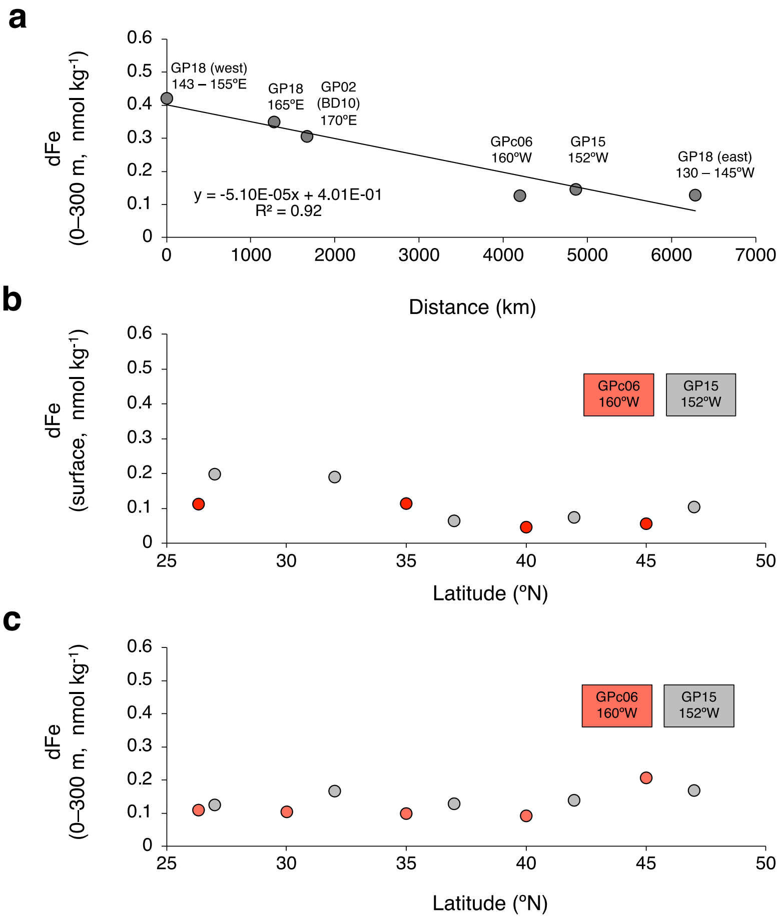


**Fig. S5.** Zonal and meridional gradients of dissolved iron (dFe) in the North Pacific. **a**) 0–300 m inventories of dFe (plotted as the 0–300 m integral divided by 300 m) between 30–45°N from GEOTRACES transects: GP02 (August–October 2012), GP18 (July 2011), GPc06 (August – September 2005), and GP15 (September–October 2018). Data for all expeditions except GP15 is drawn from the GEOTRACES Intermediate Data Product 2021 (30). **b**) Surface-most measurements of dFe (within the upper 100 m) and **c**) 0–300 m dFe inventories (calculated as in panel a) from GP15 (grey, 152°W) and GPc06 (green, 160°W). For reference, springtime measurements of dFe from the Gradients cruises (April–June, 158°W) reach maximum values of ~ 0.5 nmol Fe $kg^{-1}$.

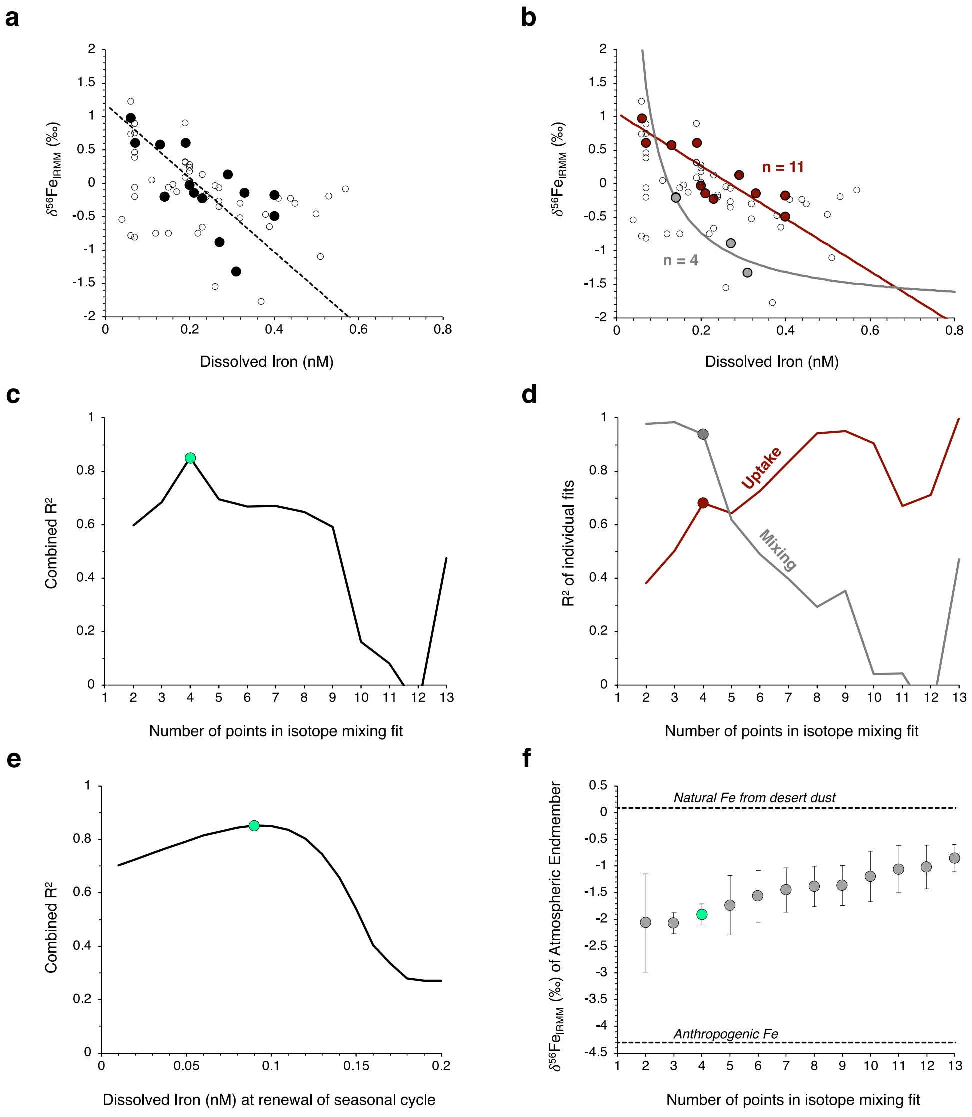


**Fig. S6.** Results from dual-curve fitting of dissolved iron (dFe) and $\delta^{56}$Fe data. **a**) Initial fitting of binned measurements (black dots, open circles show individual measurements) with a Type-II linear regression ($R^2 = 0.27$) was applied initially in order to rank residuals. Negative residuals were progressively fit by a two-component isotope mixing equation using a non-linear fit to Eq. 2. **b**) The final result of the regression retained 11 points in the open-system fractionation regime (red circles, fit by the red line) and 4 points in the isotope mixing regime (grey circles and grey line). **c**) The maximum in the combined $R^2$ (green circle) was used to identify the optimal number of points assigned to each fitting regime and (**d**) is reflective of a trade-off in the $R^2$ associated with the uptake and mixing functions, individually. **e**) Data were fit under a range of values for the background dFe concentration at the renewal of the seasonal cycle, with a value of 0.09 nM resulting in the best fit. **f**) The impact of the number of points fit by the isotope mixing equation has a small impact on the inferred isotopic composition of the atmospheric endmember, although the uncertainty associated with the fit increases substantially when 5 or more points are included. Conceptually, this reflects the distorting effects of biological fractionation on the inferred endmember value.

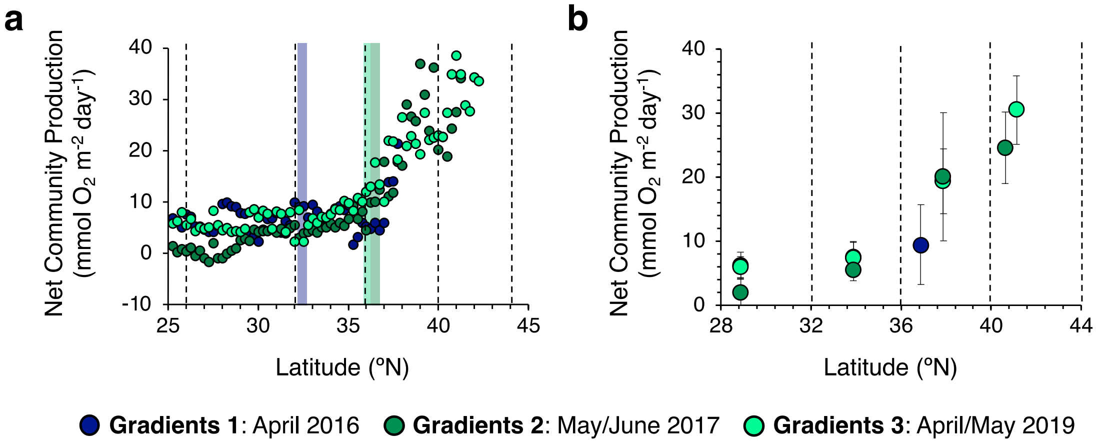


**Fig. S7.** Net community production (mmol $O_2$ $m^{-2}$ $day^{-1}$) derived from underway $O_2$/Ar measurements during the 3 Gradients cruises, described by Juranek et al. (10): **a**) as 0.5 ° averages, **b**) as mean and standard deviation after grouping into coarser latitude bins (see Fig. S1).

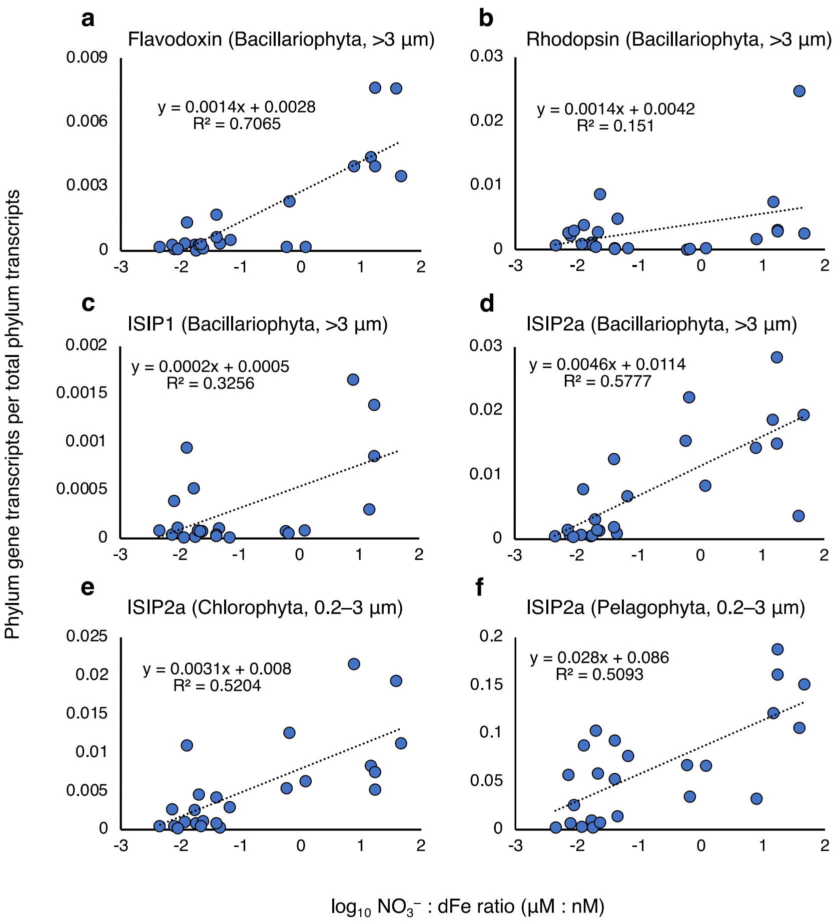


**Fig. S8.** Relationship between expression of iron stress genes (see Fig. 4 of the main text) and the ratio of nitrate ($NO_3^-$) to dissolved iron (dFe). For each cruise, dFe and $NO_3^-$ were grouped and averaged within 1° latitudinal bins and matched with transcriptome samples.

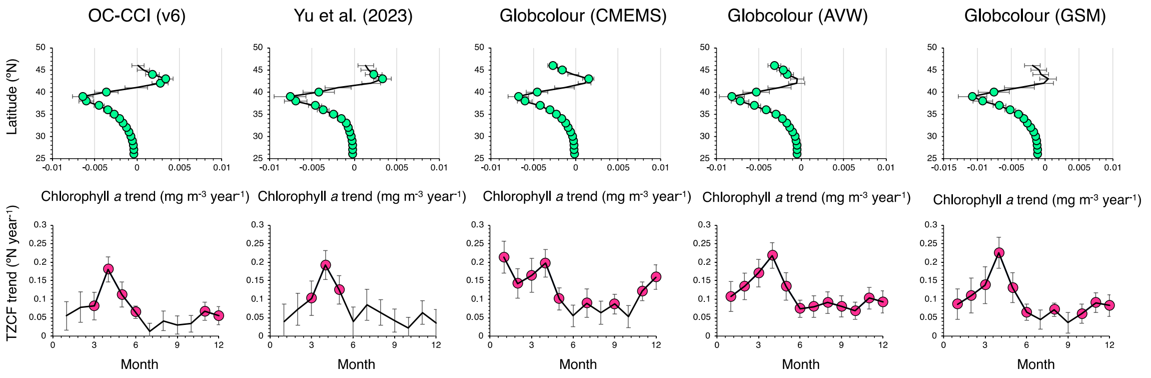


**Fig. S9.** Comparison of trends from merged satellite chlorophyll *a* products in the central NPTZ, 140–180 °W. Top panel shows slopes of linear regressions of monthly chlorophyll *a* concentration anomalies for 1° latitudinal bands during March, April, May, and June. Bottom panel shows the trend in the TZCF latitude for each month. In all panels, error bars reflect the standard error of the regressions and colored circles identify statistically significant regressions ($p < 0.05$).

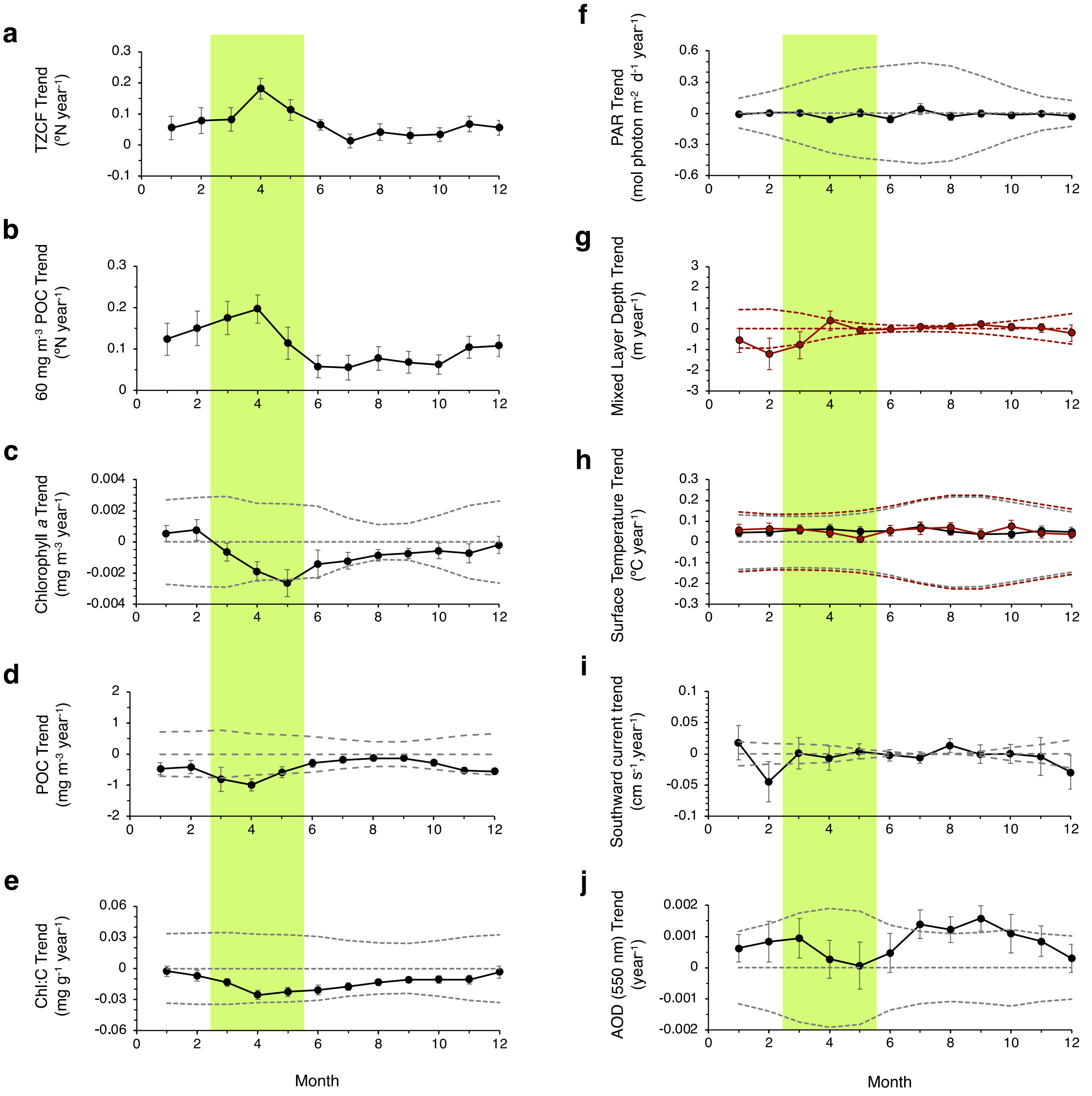


**Fig. S10.** Linear trends in biological and environmental parameters in the NPTZ based on satellite data and *in situ* observations. Parameters and symbols are the same as Fig. S3, with black lines representing satellite measurements and red lines indicating *in situ* measurements from float data (surface temperature, mixed layer depth). Regression were performed by month with mean values between 32–44 °N and 140–180 °W. In all plots, the standard error of the linear trend is shown as error bars for each month, with dotted lines indicating a ± 1 % change relative to the monthly mean value, for reference.

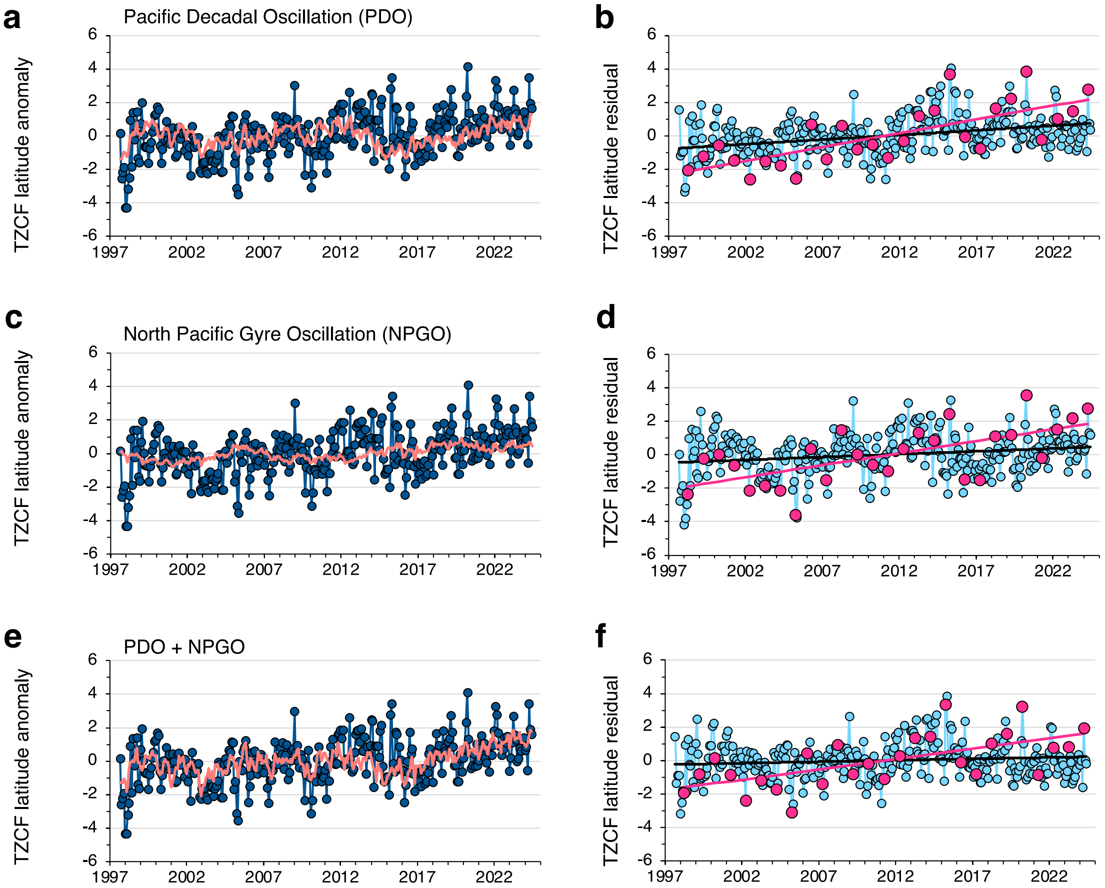


**Fig. S11.** Anomalies in the TZCF latitude and their relation to climate indices. Left panels show standardized anomalies in the TZCF (blue circles) after subtraction of monthly means. Red lines reflect best-fit regression based on optimal lag correlations between TZCF anomalies and the PDO (**a**,**b**), and NPGO (**c**,**d**), and a multiple linear regression of both indices (**e**,**f**). Right panels show residuals (light blue) of the best-fit lines and monthly TZCF anomalies plotted in the left panels. Anomalies for April (large pink circles) have the largest trend of any month. Linear trends of residuals for all months (black lines) and the month of April (pink lines) are plotted. All trends shown here were identified as statistically significant ($p < 0.05$), see Table S2.

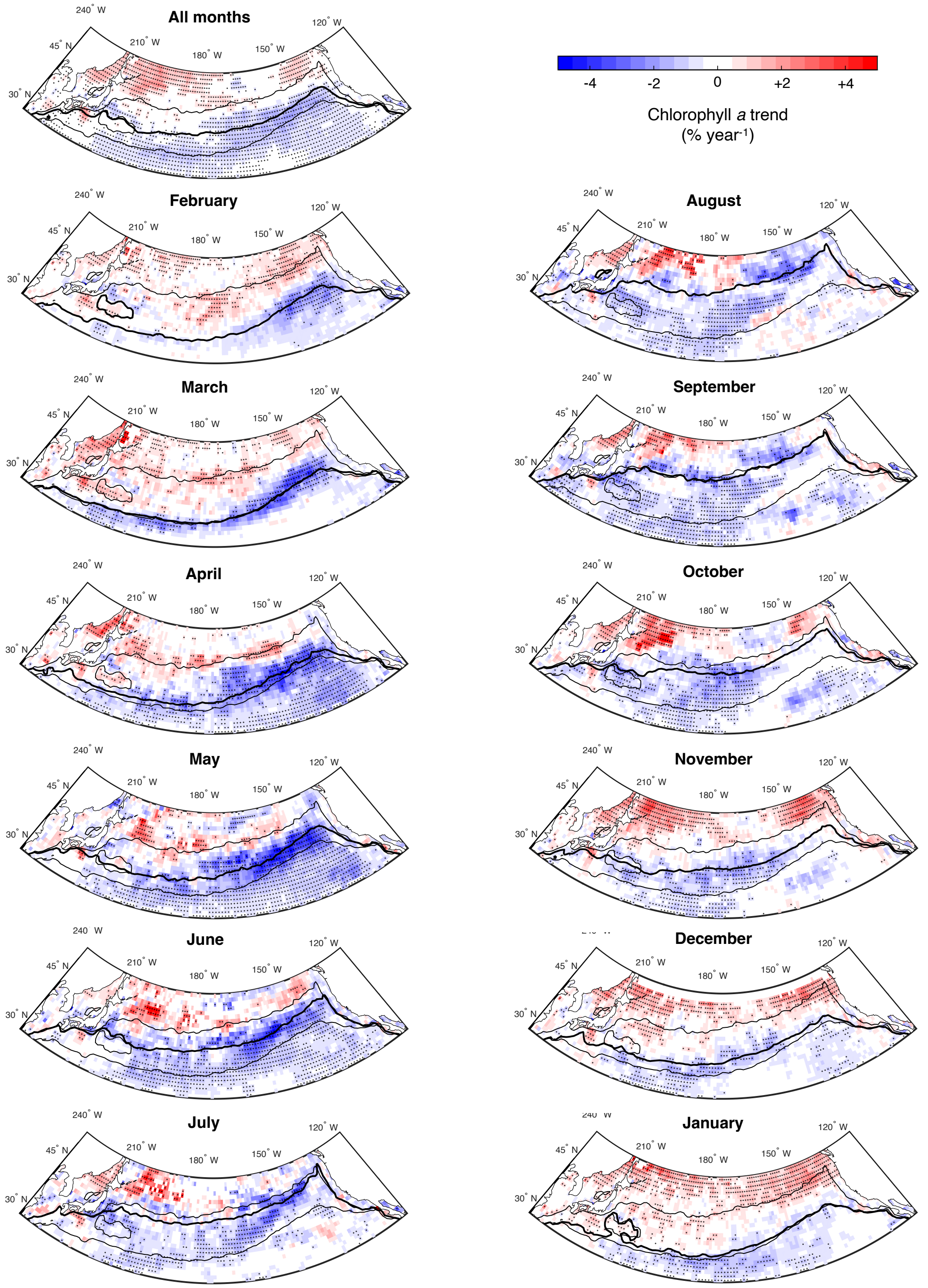


**Figure S12.** Satellite chlorophyll trends across the North Pacific for the entire timeseries and for each month. The thin lines common to all plots indicate the TZCF in February (annual minimum) and August (annual maximum), with the bold line indicating the mean TZCF position for that month. Stipples denote trends that are significantly different from 0, as indicated by $p < 0.05$ for linear regressions within 1° × 1° pixels. For the ‘All Months’ figure, only pixels with 95% coverage across all months are included.

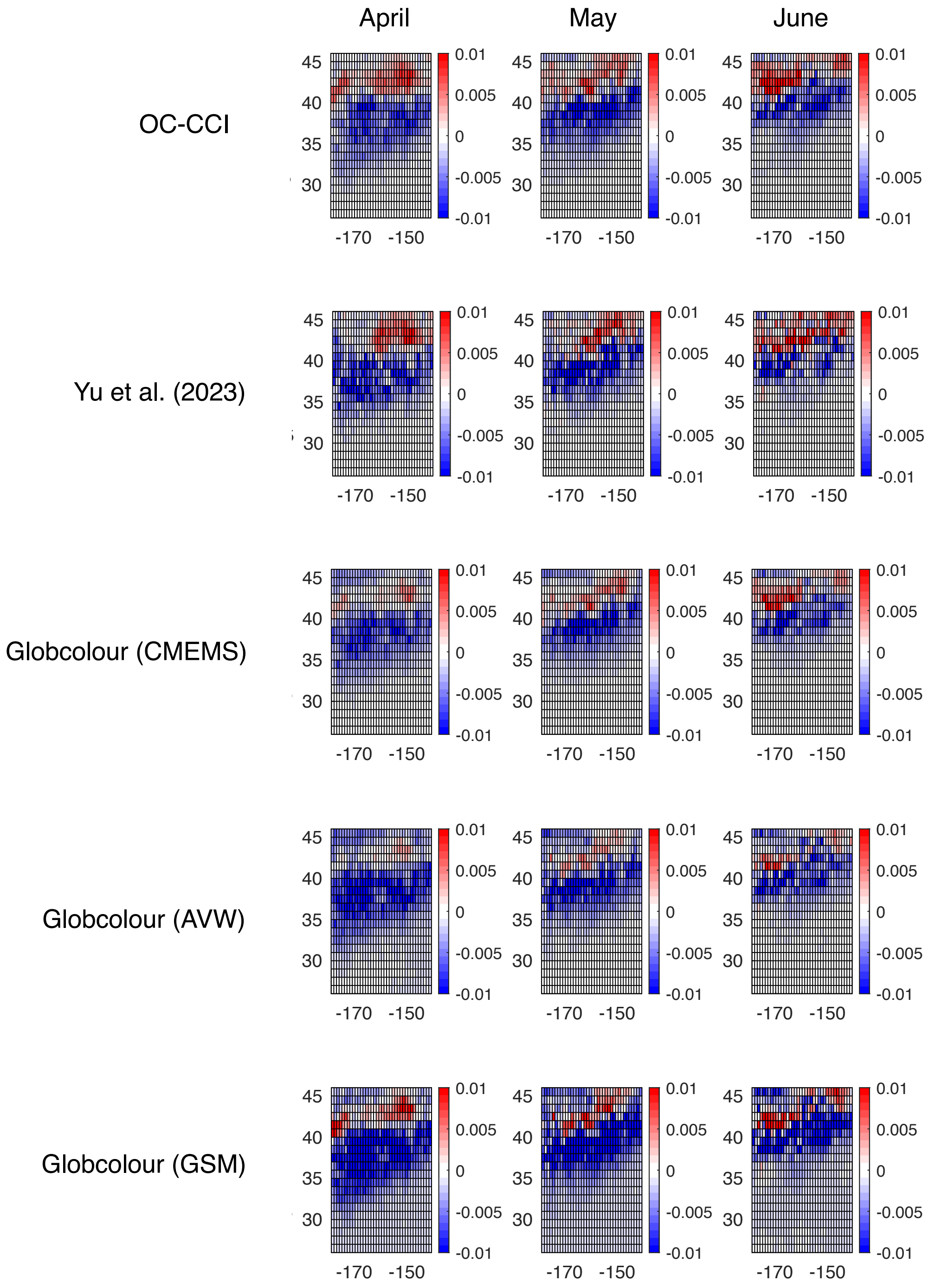


**Fig. S13.** Maps of the linear trend in chlorophyll *a* concentration (in mg $m^{-3}$ $year^{-1}$) from different merged satellite products in the central NPTZ (140–180 °W, 32–46 °N). See Fig. S9 for zonal averages and significance testing.

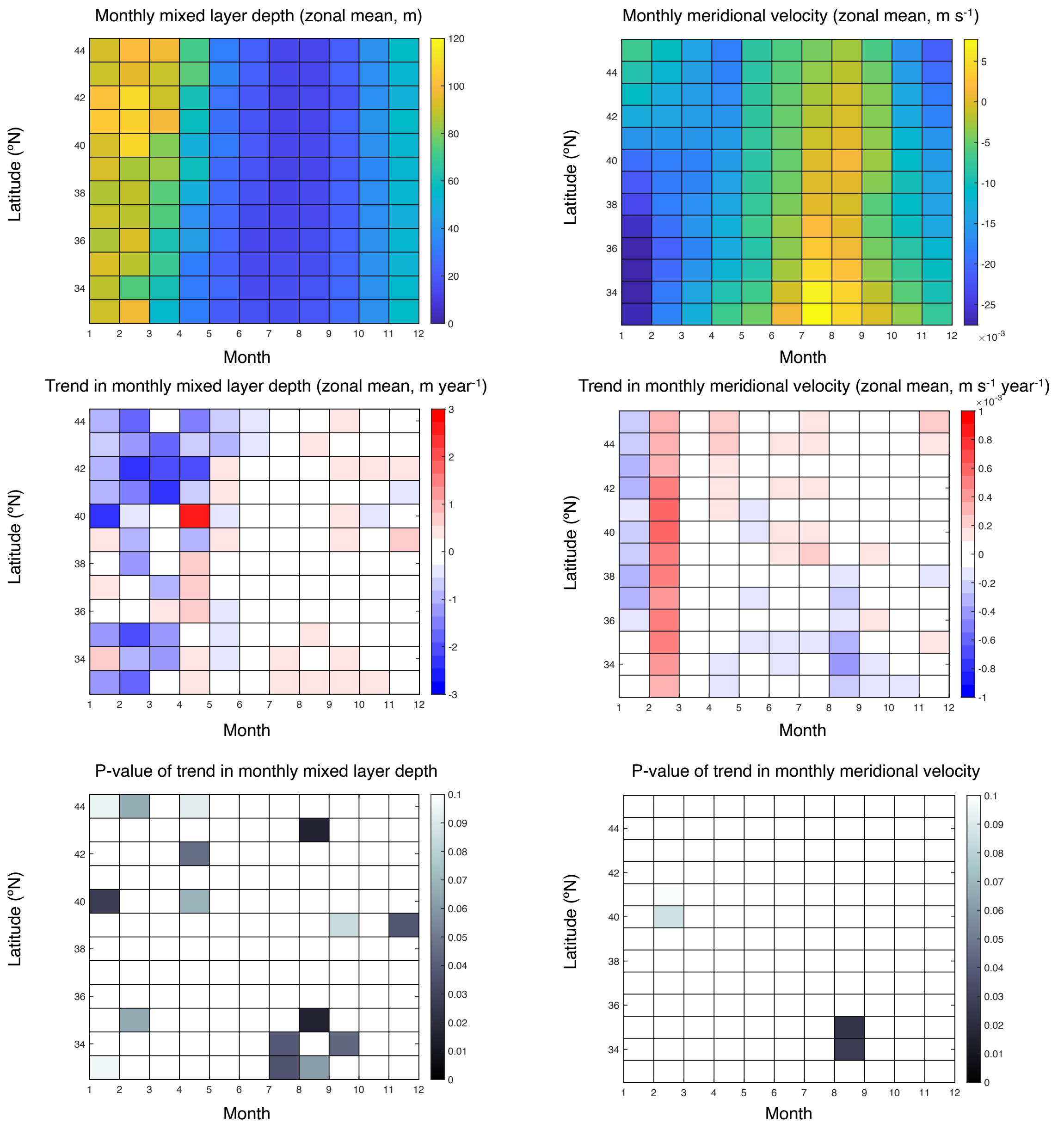


**Fig. S14.** Lack of evidence for changes in physical processes contributing to nitrate supply to the NPTZ. Mean values (top), linear trends (middle), and *p*-values (bottom) for linear regressions of monthly and 1º zonal (140–180 ºW) means of mixed layer depth (left column) and meridional surface currents (right column). Mixed layer depths derive from Argo float data from 2002 – 2022 (20). Meridional surface currents are derived from OSCAR surface current fields between 1993 – 2020 (22).

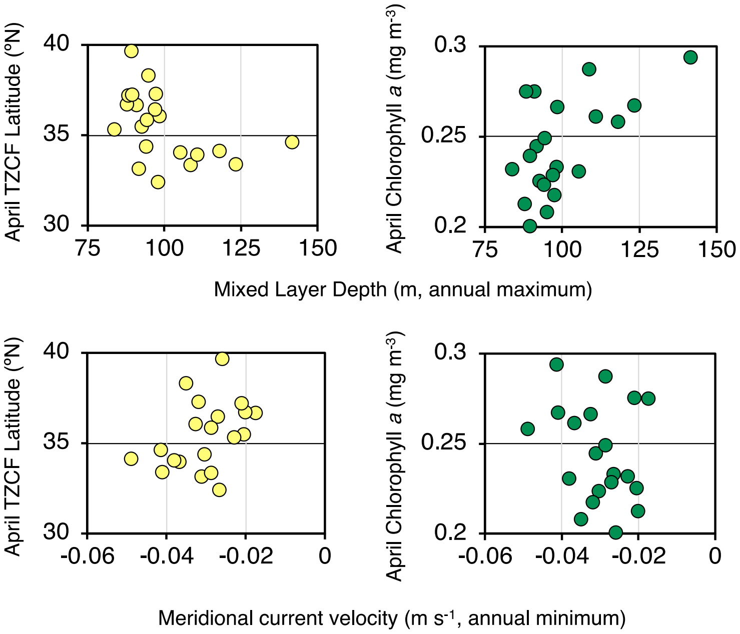


**Fig. S15.** Comparison of April TZCF latitude (yellow) and mean chlorophyll concentration (green) with annual maximum mixed layer depth (top row, 2002–2022) and the annual minimum (most southernly) surface current velocity (bottom row, 1998–2020).

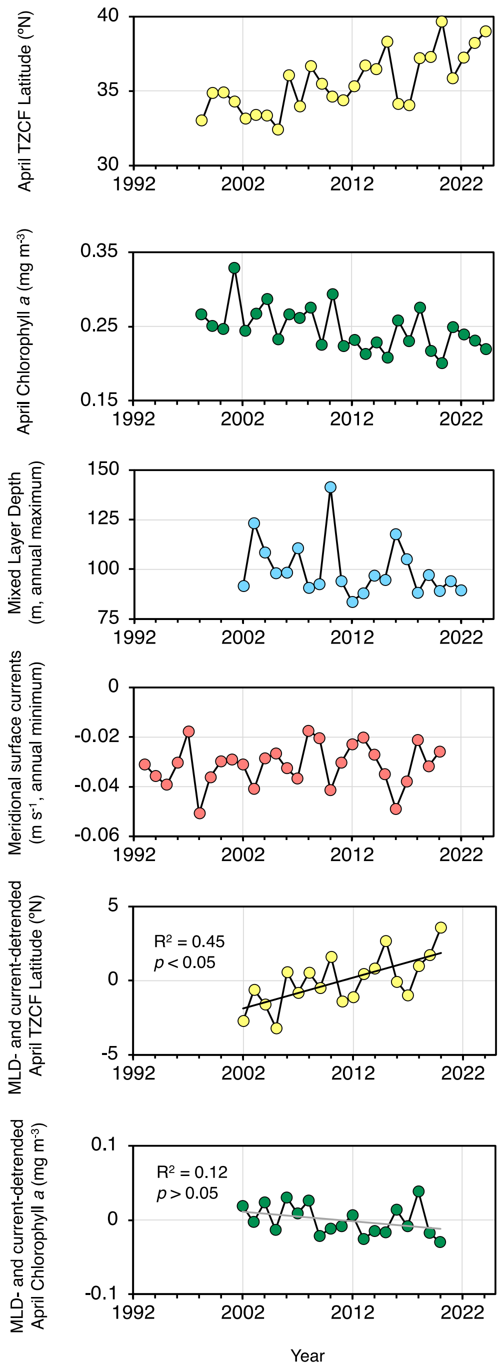


**Fig. S16.** Comparison of interannual variations in the April TZCF latitude (yellow) and mean chlorophyll *a* concentration (green) with peak mixed layer depth (MLD, blue) and meridional surface current velocity (red, note that negative values indicate prevailing southward currents). The bottom two panels show residuals of multiple linear regressions for TZCF latitude and chlorophyll *a* concentration as a function of MLD and meridional surface current velocity. Significant correlations were observed between peak MLD and chlorophyll *a*, such that the trend of residual chlorophyll *a* with time was no longer significant ($p > 0.05$). No significant relationship between April TZCF latitude was found for MLD and southward current velocity and the residuals retained a significant trend with time ($p < 0.05$).

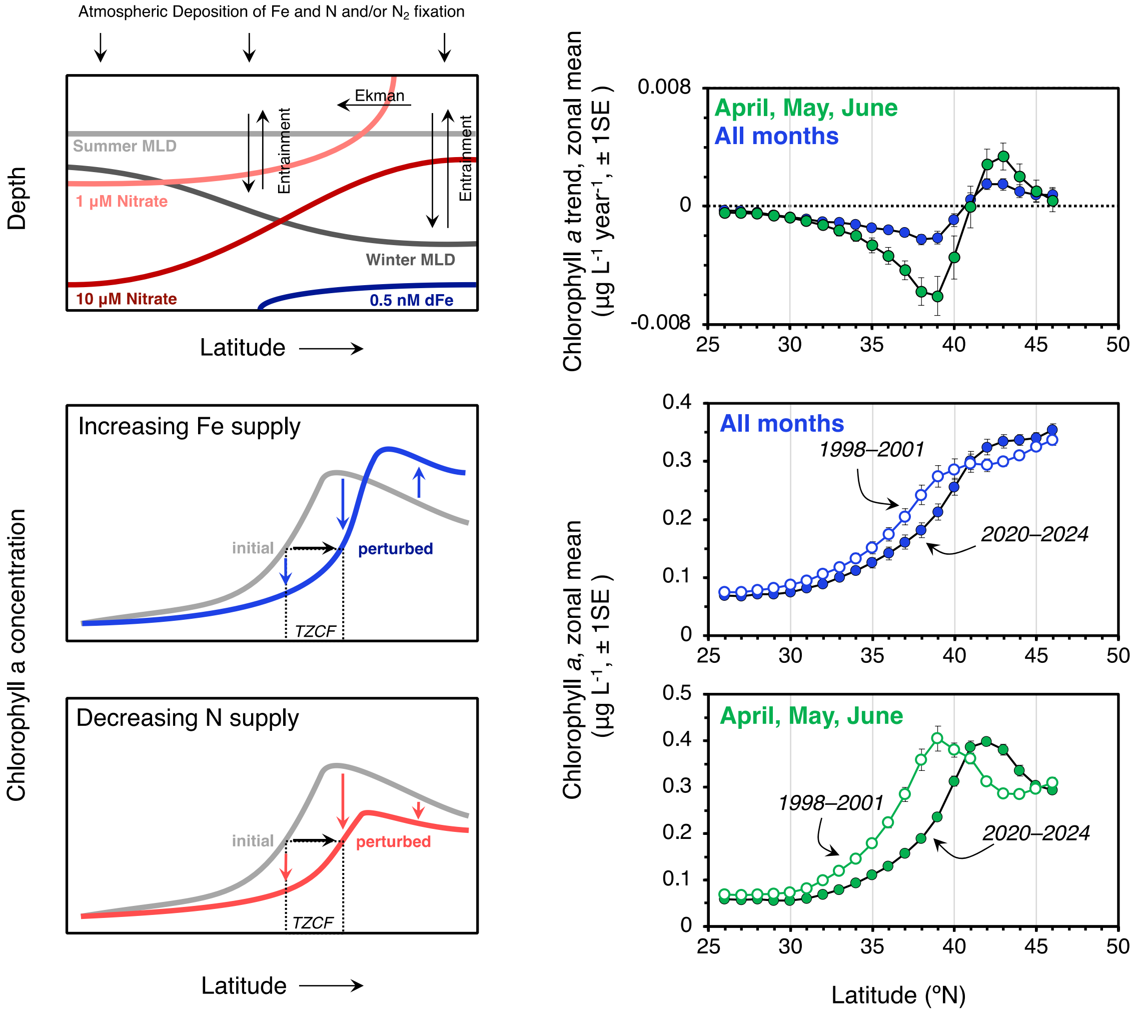


**Fig. S17.** Conceptual model of N and Fe supply and seasonal migration of the TZCF (left column) showing divergent responses in chlorophyll *a* concentration north and south of the TZCF upon increased Fe supply, but decreasing chlorophyll a both north and south of the TZCF upon decreasing N supply. Upper right panel shows the chlorophyll *a* trend in the NPTZ between 1998 – 2024 (OC-CCI) for the spring bloom period (green) and all months (blue). The latitudinal pattern of chlorophyll concentration from the start (1998–2001, open circles) and end of the timeseries (2020–2024, filled circles) is shown for all months (right, middle), and the spring bloom period (right, bottom). Note that the uncertainty in these plots reflects the standard error of linear regressions (top) or the mean value (middle, bottom).

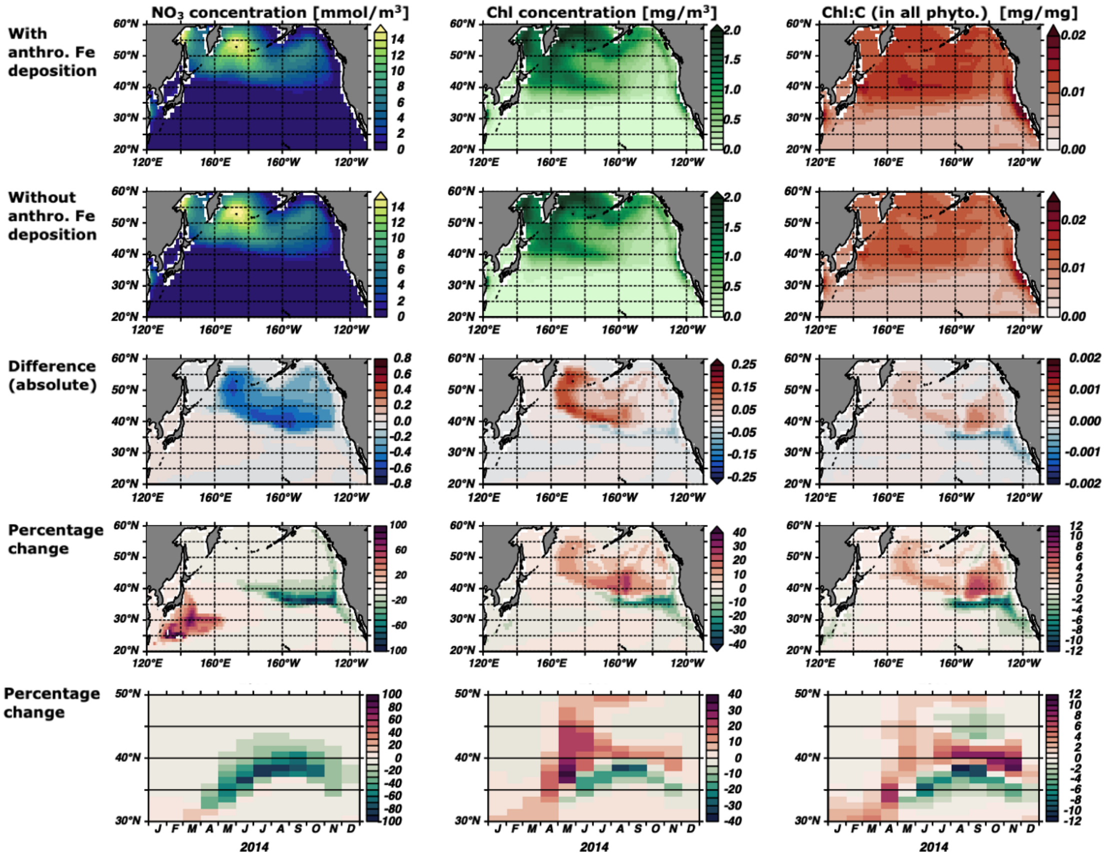


**Fig. S18.** Simulated responses of North Pacific biogeochemistry to anthropogenic Fe during late spring (June) in the PISCES biogeochemical model (31). Declines in surface nitrate, and poleward increases and equatorward decreases in chlorophyll (Chl) and the phytoplankton Chl:C ratio (summed for both phytoplankton functional types in the model) are observed across the NPTZ. In the model simulations, Fe deposition from anthropogenic and natural sources was simulated using deposition fields from an atmospheric aerosol model, covering the 1980-2014 period. The bottom panels show the last year of simulation (2014), where the impact of anthropogenic Fe deposition was largest (note that ecosystem seasonality in the model lags observations by 1–2 months, compare with Fig. S12). The simulations were set up using climatological physical forcing fields, i.e., a mean state of ocean circulation without interannual variability (32, 33). Full details of model simulation can be found in König et al. (31).

**Table S1.** Results of a two-way ANOVA testing the effect of mean dissolved iron (dFe) and mean nitrate ($NO_3^-$) on net community production (NCP) during the 3 Gradients cruises. High-resolution NCP data between 32–44 °N were grouped into 4° latitudinal bins, with corresponding mean dFe and $NO_3^-$ values assigned to each bin (see Figs. S1 and S7). The ANOVA was performed using MATLAB's *anovan* function with an interactive model, treating mean dFe and mean $NO_3^-$ as continuous predictors. The table reports the sum of squares (Sum Sq.), degrees of freedom (d.f.), mean square error (Mean Sq.), F-statistic (F), and *p*-value.

| Source | Sum Sq. | d.f. | Mean Sq. | F | *p*–value (Prob > F) |
|---|---|---|---|---|---|
| Mean dFe | 192.1 | 1 | 192.1 | 3.97 | **0.0492** |
| Mean $NO_3^-$ | 497.4 | 1 | 497.4 | 10.29 | **0.0018** |
| Mean dFe * Mean $NO_3^-$ | 851.9 | 1 | 851.9 | 17.62 | **0.0001** |
| Error | 4448.0 | 92 | 48.3 | | |
| Total | 7759.2 | 95 | | | |

**Table S2.** Linear trend in the Transition Zone Chlorophyll Front (in °N per year) between 140–180 °W based on the OC-CCI v6 product (9/1997–6/2024), after removal of the seasonal cycle and subsequent removal of lagged correlations to the Pacific Decadal Oscillation (PDO), North Pacific Gyre Oscillation (NPGO), and PDO+NPGO. Bold, red values indicate slopes determined to be significantly different from 0 (*p*-values < 0.05).

| Month | Seasonal cycle removed [a] | | PDO removed [b] | | NPGO removed [c] | | PDO + NPGO removed [d] | |
|---|---|---|---|---|---|---|---|---|
| | Slope | $R^2$ | Slope | $R^2$ | Slope | $R^2$ | Slope | $R^2$ |
| 1 | 0.055 | 0.08 | 0.037 | 0.05 | 0.025 | 0.02 | 0.003 | 0.00 |
| 2 | 0.078 | 0.13 | 0.058 | 0.10 | 0.031 | 0.02 | 0.006 | 0.00 |
| 3 | **0.080** | **0.15** | **0.056** | **0.13** | 0.040 | 0.04 | 0.012 | 0.01 |
| 4 | **0.181** | **0.54** | **0.165** | **0.54** | **0.144** | **0.42** | **0.125** | **0.39** |
| 5 | **0.119** | **0.36** | **0.106** | **0.31** | **0.086** | **0.22** | **0.070** | **0.16** |
| 6 | **0.065** | **0.38** | **0.053** | **0.28** | 0.030 | 0.09 | 0.014 | 0.03 |
| 7 | 0.011 | 0.01 | 0.008 | 0.01 | -0.021 | 0.03 | -0.026 | 0.05 |
| 8 | 0.041 | 0.09 | 0.032 | 0.06 | 0.008 | 0.00 | -0.003 | 0.00 |
| 9 | 0.031 | 0.06 | 0.019 | 0.02 | 0.003 | 0.00 | -0.011 | 0.01 |
| 10 | 0.034 | 0.08 | 0.026 | 0.09 | 0.008 | 0.01 | -0.002 | 0.00 |
| 11 | **0.067** | **0.22** | **0.052** | **0.27** | 0.033 | 0.07 | 0.014 | 0.03 |
| 12 | **0.056** | **0.17** | **0.039** | **0.15** | 0.015 | 0.02 | -0.005 | 0.00 |
| All | **0.069** | **0.17** | **0.055** | **0.14** | **0.034** | **0.05** | **0.017** | **0.02** |

[a] seasonal cycle removed by subtraction of monthly means.
[b] optimal fit at 1 month lag: z (m) = –0.4188 – 0.5354 [PDO (m – 1)]; $R^2$ = 0.21, $p = 1.8 \times 10^{-18}$.
[c] optimal fit at 1 month lag: z (m) = –0.0042 – 0.2756 [NPGO (m – 1)]; $R^2$ = 0.09, $p = 5.6 \times 10^{-8}$.
[d] optimal fit: z (m) = –0.4380 – 0.5542 [PDO (m – 1)] – 0.2980 [NPGO (m – 1)]; $R^2$ = 0.32, $p = 4.0 \times 10^{-27}$.

## SI References


1. Pinedo-González P, et al. (2020) Anthropogenic Asian aerosols provide Fe to the North Pacific Ocean. *Proc Natl Acad Sci* 117(45):27862–27868.
2. Hawco NJ, et al. (2021) Iron depletion in the deep chlorophyll maximum: mesoscale eddies as natural iron fertilization experiments. *Global Biogeochem Cycles*:e2021GB007112.
3. Hawco NJ, et al. (2020) Metal isotope signatures from lava-seawater interaction during the 2018 eruption of Kīlauea. *Geochim Cosmochim Acta* 282:340–356.
4. Conway TM, Rosenberg AD, Adkins JF, John SG (2013) A new method for precise determination of iron, zinc and cadmium stable isotope ratios in seawater by double-spike mass spectrometry. *Anal Chim Acta* 793:44–52.
5. Sieber M, et al. (2023) Biological, physical, and atmospheric controls on the distribution of cadmium and its isotopes in the Pacific Ocean. *Global Biogeochem Cycles* 37(2):e2022GB007441.
6. Sieber M, et al. (2023) The importance of reversible scavenging for the marine Zn cycle evidenced by the distribution of zinc and its isotopes in the Pacific Ocean. *J Geophys Res Ocean* 128(4):e2022JC019419.
7. Lanning NT, et al. (2023) Isotopes illustrate vertical transport of anthropogenic Pb by reversible scavenging within Pacific Ocean particle veils. *Proc Natl Acad Sci* 120(23):e2219688120.
8. Sieber M, et al. (2021) Isotopic fingerprinting of biogeochemical processes and iron sources in the iron-limited surface Southern Ocean. *Earth Planet Sci Lett* 567:116967.
9. Hunt HR, et al. (2022) Distinguishing the influence of sediments, the Congo River, and water-mass mixing on the distribution of iron and its isotopes in the Southeast Atlantic Ocean. *Mar Chem* 247:104181.
10. Juranek LW, et al. (2020) The importance of the phytoplankton “middle class” to ocean net community production. *Global Biogeochem Cycles* 34(12):e2020GB006702.
11. Kurisu M, Takahashi Y, Iizuka T, Uematsu M (2016) Very low isotope ratio of iron in fine aerosols related to its contribution to the surface ocean. *J Geophys Res Atmos* 121(18):11–119.
12. Kurisu M, Sakata K, Uematsu M, Ito A, Takahashi Y (2021) Contribution of combustion Fe in marine aerosols over the northwestern Pacific estimated by Fe stable isotope ratios. *Atmos Chem Phys* 21(20):16027–16050.
13. Kurisu M, Adachi K, Sakata K, Takahashi Y (2019) Stable isotope ratios of combustion iron produced by evaporation in a steel plant. *ACS Earth Sp Chem* 3(4):588–598.
14. Hayes CT, et al. (2015) Thorium isotopes tracing the iron cycle at the Hawaii Ocean Time-series Station ALOHA. *Geochim Cosmochim Acta* 169:1–16.
15. Hawco NJ, et al. (2022) Recycling of dissolved iron in the North Pacific Subtropical Gyre. *Limnol Oceanogr* 67(11):2448–2465.
16. Di Lorenzo E, et al. (2008) North Pacific Gyre Oscillation links ocean climate and ecosystem change. *Geophys Res Lett* 35(8).
17. Polovina JJ, Howell E, Kobayashi DR, Seki MP (2001) The transition zone chlorophyll front, a dynamic global feature defining migration and forage habitat for marine resources. *Prog Oceanogr* 49(1–4):469–483.
18. Ayers JM, Lozier MS (2010) Physical controls on the seasonal migration of the North Pacific transition zone chlorophyll front. *J Geophys Res Ocean* 115(C5).
19. Follett CL, Dutkiewicz S, Forget G, Cael BB, Follows MJ (2021) Moving ecological and biogeochemical transitions across the North Pacific. *Limnol Oceanogr* 66(6):2442–2454.
20. Holte J, Talley LD, Gilson J, Roemmich D (2017) An Argo mixed layer climatology and database. *Geophys Res Lett* 44(11):5618–5626.
21. Holte J, Talley L (2009) A new algorithm for finding mixed layer depths with applications to Argo data and Subantarctic Mode Water formation. *J Atmos Ocean Technol* 26(9):1920–1939.

22. Dohan K (2021) Ocean surface current analyses real-time (OSCAR) surface currents–final 0.25 degree (version 2.0).
23. Bonjean F, Lagerloef GSE (2002) Diagnostic model and analysis of the surface currents in the tropical Pacific Ocean. *J Phys Oceanogr* 32(10):2938–2954.
24. Henderikx Freitas F, White AE, Quay PD (2020) Diel measurements of oxygen-and carbon-based ocean metabolism across a trophic gradient in the North Pacific. *Global Biogeochem Cycles* 34(11):e2019GB006518.
25. Sunda WG, Huntsman SA (1995) Iron uptake and growth limitation in oceanic and coastal phytoplankton. *Mar Chem* 50(1–4):189–206.
26. Strzepek RF, Boyd PW, Sunda WG (2019) Photosynthetic adaptation to low iron, light, and temperature in Southern Ocean phytoplankton. *Proc Natl Acad Sci* 116(10):4388–4393.
27. Marchetti A, et al. (2009) Ferritin is used for iron storage in bloom-forming marine pennate diatoms. *Nature* 457(7228):467.
28. Prospero JM, Savoie DL, Arimoto R (2003) Long-term record of nss-sulfate and nitrate in aerosols on Midway Island, 1981–2000: Evidence of increased (now decreasing?) anthropogenic emissions from Asia. *J Geophys Res Atmos* 108(D1):AAC-10.
29. Boyd PW, et al. (2005) FeCycle: Attempting an iron biogeochemical budget from a mesoscale SF6 tracer experiment in unperturbed low iron waters. *Global Biogeochem Cycles* 19(4).
30. NERC. The GEOTRACES Intermediate Data Product 2021 (IDP2021) (2021). *NERC EDS Br Oceanogr Data Cent NOC*. doi: 10.5285/cf2d9ba9-d51d-3b7c-e053-8486abc0f5fd .
31. König D, Conway TM, Hamilton DS, Tagliabue A (2022) Surface ocean biogeochemistry regulates the impact of anthropogenic aerosol Fe deposition on the cycling of iron and iron isotopes in the North Pacific. *Geophys Res Lett* 49(13):e2022GL098016.
32. Hamilton DS, et al. (2020) Recent (1980 to 2015) trends and variability in daily-to-interannual soluble iron deposition from dust, fire, and anthropogenic sources. *Geophys Res Lett* 47(17):e2020GL089688.
33. Rathod SD, et al. (2020) A mineralogy-based anthropogenic combustion-iron emission inventory. *J Geophys Res Atmos* 125(17):e2019JD032114.

**Dataset S1 (separate file).** Near-surface dissolved iron and iron isotope data from the Gradients 1, 2, and 3 expeditions and the GP15 expedition used in this study.
